# Synthesis of Novel Biologically Active Proflavine Ureas Designed on the Basis of Predicted Entropy Changes

**DOI:** 10.3390/molecules26164860

**Published:** 2021-08-11

**Authors:** Ladislav Janovec, Eva Kovacova, Martina Semelakova, Monika Kvakova, Daniel Kupka, David Jager, Maria Kozurkova

**Affiliations:** 1 Department of Organic Chemistry, Faculty of Science, P. J. Safarik University in Kosice, Moyzesova 11, 040 01 Kosice, Slovakia; evakovacova2702127@gmail.com; 2 Department of Medical Biology, Faculty of Medicine, P. J. Safarik University in Kosice, Trieda SNP1, 040 11 Kosice, Slovakia; martina.semelakova@upjs.sk; 3 Department of Experimental Medicine, Faculty of Medicine, P. J. Safarik University in Kosice, Trieda SNP1, 040 11 Kosice, Slovakia; monika.kvakova@upjs.sk; 4 Institute of Geotechnics, Slovak Academy of Sciences, Watsonova 45, 040 01 Kosice, Slovakia; dankup@saske.sk (D.K.); jager@saske.sk (D.J.); 5 Department of Biochemistry, Faculty of Science, P. J. Safarik University in Kosice, Moyzesova 11, 040 01 Kosice, Slovakia; maria.kozurkova@upjs.sk; 6 Biomedical Research Center, University Hospital Hradec Kralove, Sokolovska 581, 500 05 Hradec Kralove, Czech Republic

**Keywords:** proflavine ureas, molecular design, cytostatic activity, cytotoxicity

## Abstract

A novel series of proflavine ureas, derivatives **11a**–**11i**, were synthesized on the basis of molecular modeling design studies. The structure of the novel ureas was obtained from the pharmacological model, the parameters of which were determined from studies of the structure-activity relationship of previously prepared proflavine ureas bearing *n*-alkyl chains. The lipophilicity (Log*P*) and the changes in the standard entropy (Δ*S*°) of the urea models, the input parameters of the pharmacological model, were determined using quantum mechanics and cheminformatics. The anticancer activity of the synthesized derivatives was evaluated against NCI-60 human cancer cell lines. The urea derivatives azepyl **11b**, phenyl **11c** and phenylethyl **11f** displayed the highest levels of anticancer activity, although the results were only a slight improvement over the hexyl urea, derivative **11j**, which was reported in a previous publication. Several of the novel urea derivatives displayed GI_50_ values against the HCT-116 cancer cell line, which suggest the cytostatic effect of the compounds azepyl **11b**–0.44 μM, phenyl **11c**–0.23 μM, phenylethyl **11f**–0.35 μM and hexyl **11j**–0.36 μM. In contrast, the novel urea derivatives **11b**, **11c** and **11f** exhibited levels of cytotoxicity three orders of magnitude lower than that of hexyl urea **11j** or amsacrine.

## 1. Introduction

Previous studies have shown that compounds based on an acridine chromophore possess a wide range of interesting biological activities [1]. The main type of biological effect exhibited by acridine derivatives is that of antiproliferative activity [2]. The main interaction mode of acridines with biological macromolecules is an intercalation reaction with nucleic acids where an electron-deficient acridine chromophore inserts between adjacent base pairs [3,4]. This process affects cell division on the molecular and enzymatic level, leading to the suppression of further cell proliferation. The enzymatic inhibitory effect of acridine-based compounds takes the form of specific interactions with topoisomerases I/II, whereby the ability to stabilize G-rich sequences of telomeric DNA in the form of a G-quadruplex indirectly inhibits the activity of the enzyme telomerase [5].

**Figure 1:**
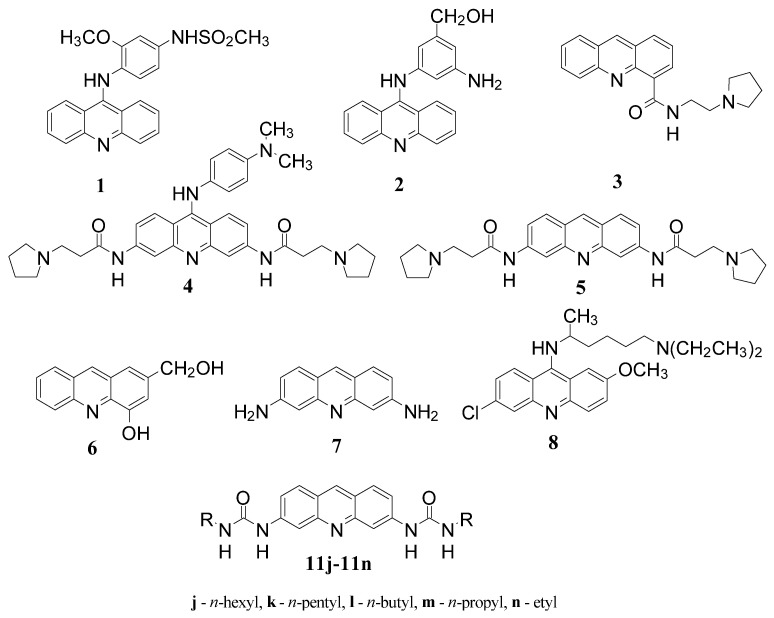
Acridine derivatives with biological activity.

Genomic DNA is densely packed and undergoes superhelical strain during the process of cell division. Two principal enzymes are involved in this process: topoisomerases I and II. Topo I breaks one strand of duplex DNA transiently and allows the rotation about the unbroken strand, while Topo II enzymes break both strands and passes one region of DNA through the resulting gap. Topoisomerases I/II cleave the phosphodiester backbone through a nucleophilic attack on a tyrosine hydroxyl group, which results in the formation of a covalent bond between the enzyme and the broken strand [6]. Compounds which are capable of interfering with the covalent enzyme–DNA complex are termed topoisomerases poisons. Two major acridine-based agents act as topoisomerase inhibitors within the cell proliferation process: amsacrine-like compounds **1** and **2** and compounds derived from the structure of DACA **3** (Figure 1) [7].

Telomerase is a special type of DNA polymerase which maintains an elongation of a telomeric DNA shortening in tumor cells after each cell division cycle. Telomerase is an RNA reverse transcriptase that synthesizes telomeric DNA from an RNA template, thereby leading to the uncontrollable proliferation of malign cells [6]. Previous studies have shown that substituted acridines such as compounds **4** and **5** exhibit the ability to interact with the telomeric DNA, resulting in interesting cytostatic activities (Figure 1) [8,9].

Recently, the total synthesis of inubosine B**6**, an acridine alkaloid that initiates neuroregeneration, has been reported (Figure 1) [10]. Antimicrobial properties are another important category of biological activity displayed by acridine derivatives [11,12]. Proflavine **7** is the example of a typical antibacterial and antifungal agent, while quinacrine **8** exhibits antimalarial properties (Figure 1) [13].

In this study, we present a pharmacological model based on the entropy changes in the urea derivatives **11j**–**11n** accompanying their DNA binding process. This pharmacological model could be used as a tool in the design of new urea-based intercalators with potential anticancer activity.

## 2. Results and Discussion

**Figure 2:**
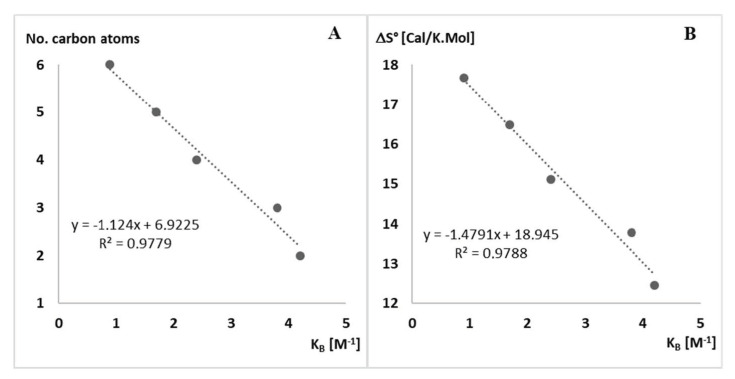
(**A**). Relationship of the binding constants *K*_B_ and the length of the alkyl chain of the urea derivatives. (**B**). Relationship of the binding constant *K*_B_ and the change in the standard entropy Δ*S*°. The binding constants *K*_B_ × 10^5^ of the *ct*DNA–urea **11j**–**11n** intercalation complexes are given as a molar concentration. A value of the change in the standard entropy Δ*S*° of urea derivatives **11j**–**11n** given in cal.mol^−1^. K^−1^ was obtained using the PM7 method. Several carbon atoms define the number of carbons in the alkyl chain of the derivative. **11j**–*n*-hexyl, **11k**–*n*-pentyl, **11l**–*n*-butyl, **11m**–*n*-propyl, **11n**–ethyl.

The stability of the intercalation between *ct*DNA and ureas **11j**–**11n** (Figure 1) is highly dependent upon the length of the urea alkyl chains. Previous studies have observed that the enlargement of alkyl chains led to the reduced *K*_B_ value of the binding complex, and vice versa (Figure 2A) [14]. 

This finding prompted us to determine the relationship of the *K*_B_ values and the Δ*S*° values for the urea derivatives **11j**–**11n** (Figure 2B). The term Δ*S*° reflects the structural features of the chains of specific urea derivatives, as is shown in Figure 2, and expresses the gap in the values of the standard entropy, *S*°, obtained at the temperatures of 300 K and of 340 K using the PM7 method (Table 1). These findings will be discussed further within the framework of the urea-based molecular model design study.

In order to predict the strength of the binding between the ligand and DNA, MM-GBSA (PBSA) calculations should be performed for specific intercalation complexes followed by a normal mode analysis to reveal the thermodynamic parameters of the binding process; more specifically, the binding free energy change and its enthalpic and entropic components [15]. Entropy changes are the consequence of the loss of degrees of freedom and enthalpy changes arise from conformation discrepancies when an intercalation complex is formed [6].

Furthermore, it is also necessary to consider the appropriate modes by which the ligand binds to DNA in order to obtain accurate results for the required calculations. However, this task is complicated by the complexity of the intermolecular interactions involved in the binding process. As a result, our study simplifies this process by employing an approach based on the following considerations.

We hypothesized that only the chains of ureas **11j**–**11n** undergo structural changes during the formation of the complex. This idea arose from our expectation that the flexible alkyl chains would adapt to the macromolecule during the binding process rather than vice versa. We further hypothesized that a DNA lattice could undergo similar changes during the formation of complexes with urea derivatives **11j**–**11n**. Given such hypotheses, we could expect that the enthalpy changes in intercalation reactions across the entire series of urea derivatives **11j**–**11n** would not differ substantially; therefore, ΔΔ*H*_rxn_ = ~0.

On such a basis, it is possible to suggest that the stability of the binding complex depends substantially on the entropy changes in derivatives **11j**–**11n** which accompany an intercalation reaction. This can be rationalized using the above-mentioned relationship of *K*_B_ values versus the Δ*S*° values (Figure 2B). In terms of structure, the Δ*S*° value reflects the structural features of the chains of derivatives **11j**–**11n**. By extending this relationship as a prediction tool, the Δ*S*° value could predict how strongly the urea model can bind to DNA. 

**Figure 3:**
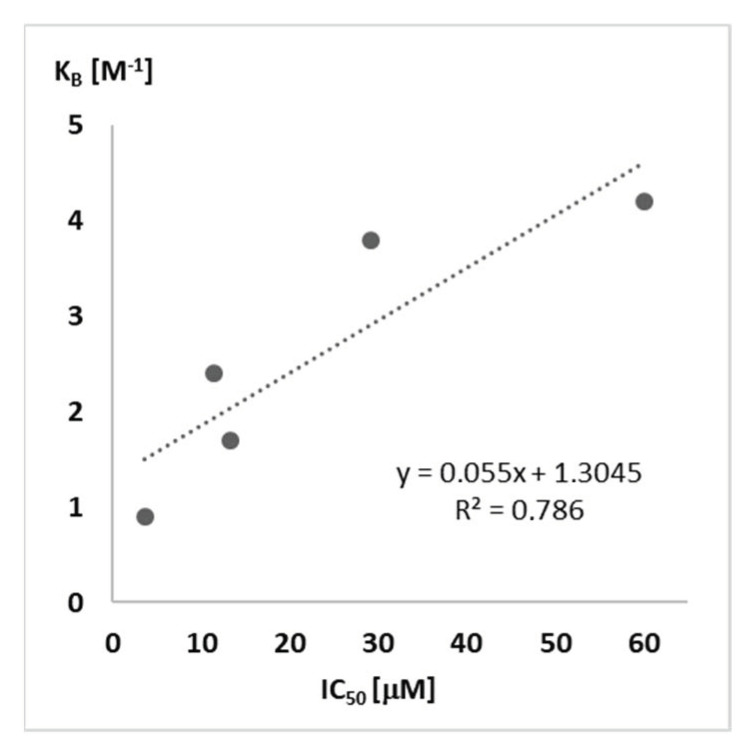
Relationship of the inhibition concentration IC_50_ and the binding constant *K*_B_. The binding constant *K*_B_ × 10^5^ of the intercalation between ureas **11j**–**11n** and *ct*DNA is given as a molar concentration. The value of the inhibition concentration IC_50_ of ureas **11j**–**11n** against HeLa cancer cell lines is given as a micromolar concentration.

**Figure 4:**
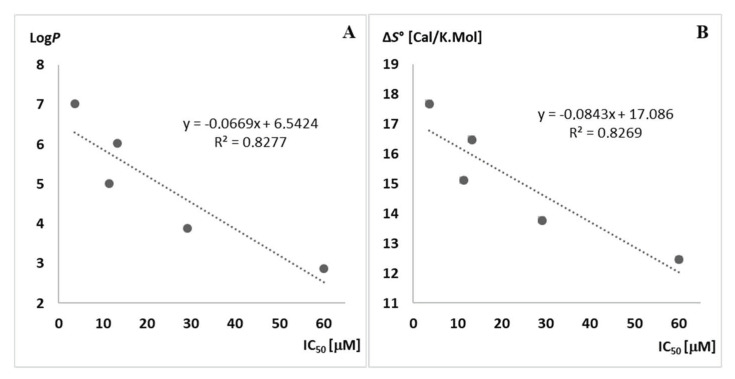
(**A**). Relationship of the inhibition concentration IC_50_ and the lipophilicity Log*P*. (**B**). Relationship of the inhibition concentration IC_50_ and the change in the standard entropy Δ*S*°. The value of the lipophilicity Log*P* was determined using the Molinspiration software program (molinspiration.com). The value of the inhibition concentration IC_50_ of ureas **11j**–**11n** against HeLa cancer cell lines is given as a micromolar concentration. The value of the change in the standard entropy Δ*S*° of ureas **11j**–**11n** given in cal.mol^−1^. K^−1^ was obtained using the PM7 method.

Unexpectedly, the cytotoxicity (IC_50_) of ureas **11j**–**11n** was not found to increase with the increasing of the binding constant, *K*_B_, a finding which contradicts the hypothesis that DNA is the typical pharmacological target of such substances (Figure 3). A positive correlation was found for the relationship of cytotoxicity (IC_50_) and lipophilicity (Log*P*), which thereby reflects the fact that the ability of specific urea derivatives to penetrate the cell membrane is of equal importance in the anticancer activity as its ability to bind to DNA (Figure 4A). Analogously, the prediction of the lipophilicity of a given urea model may offer a useful estimate of its anticancer potential. The task which we addressed was, therefore, how to utilize the two opposing physicochemical properties of log*P* and *K*_B_ to predict the biological activity of a specific urea model.

Bearing in mind the facts outlined above, it is clear that it would not be possible to design a urea-based model that exhibits both strong DNA binding capabilities and high levels of lipophilicity. One solution, therefore, would be to find a compromise structure, a molecule with sufficient lipophilicity to penetrate a cell membrane but which could still be able to form a stable complex with DNA. For this purpose, a pharmacological model was developed which utilized the relationship of IC_50_ versus Log*P* and the relationship of *K*_B_ versus Δ*S*° as prediction tools, both of which were discussed above. According to this model, only a determination of the value of log*P* and the value of Δ*S*° would be required to determine the binding capacity and anticancer activity for a given urea model. 

The pharmacological model is based on a scoring system that determines a specific range of score values which urea derivative models must achieve before they would be considered for synthesis. The score value is the sum of the log*P* value and the Δ*S*° value of the urea model and the acceptable range for the derivative models was derived from the score values for the urea derivatives hexyl **11l** and butyl **11n**, compounds which display a reasonable range of biological activity (Figure 4). 

**Figure 5:**
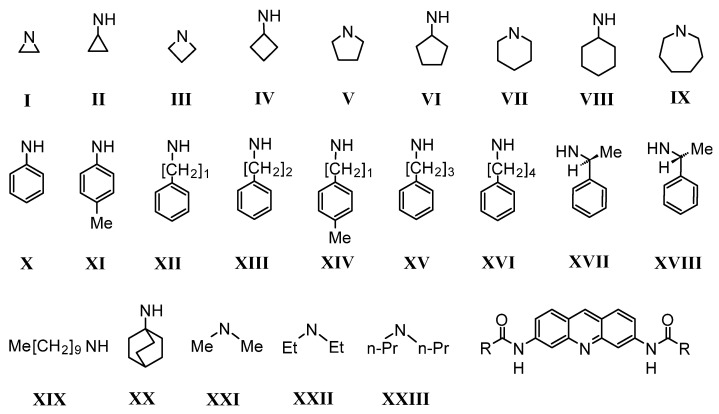
Urea models **I**–**XXIII** used within the molecular modeling study.

In order to identify potential new urea-based intercalators with increased biological activity, quantum mechanics and cheminformatics were used to obtain the log*P* and Δ*S*° values for specific urea models. Urea models I–XXIII were then modelled using ChemSketch software [16] (Figure 5).

The optimization of models I–XXIII were performed using the PM7 method and a post processing normal mode analysis provided the values of standard entropy, *S*° (Table 2). The calculations were performed using the MOPAC2016 software package, while the logP values for the model structures were obtained using Molinspiration online software [17,18]. The logP and Δ*S*° values were summed together in order to provide the score values of urea models I–XXIII (Table 2). Specific models would be considered as the leading structures if their score value fell within the range defined by the values of 20.1 and 24.7. Only models which featured six carbon atoms in their chains were synthesized: models VIII–XIV, XVII and XVIII (Figure 5).

**Scheme 1:**
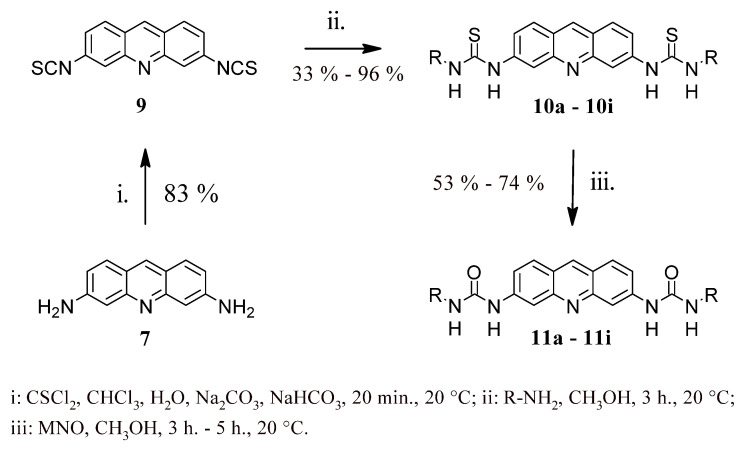
Synthesis of ureas **11a**–**11i**.

Models VIII–XIV, XVII and XVIII were used as the basis for the synthesis of urea derivatives **11a**–**11i**, which were prepared following our previously published procedure (Scheme 1). The synthesis of 3,6-diisothiocyanatoacridine (**9**) was improved substantially, with the yield of the reaction increasing from the previous 53% to 83%. Isothiocyanate **9** was purified through crystallization from toluene and was subsequently used in the synthesis of the related thioureas **10a**–**10i** which were isolated as light yellow solids. The next step of the process was the preparation of the final urea derivatives **11a**–**11i** through the reaction of the related thioureas with mesitylnitrile oxide in methanol. The synthesized derivatives were then used in the subsequent biological and biochemical experiments.

Derivatives **11a**–**11i** were subjected to in vitro screening on cancer cell lines conducted by the Developmental Therapeutic Program of the National Cancer Institute (NCI). A one-dose screen of each derivative against NCI-60 panels consisting of sixty human cancer cell lines was performed in order to identify the derivatives which exhibited the highest biological activity. On the basis of these results, ureas **11b**, **11c** and **11f** were selected for further evaluation of their growth inhibition activity. From the panel of sixty human cancer cell lines, a single cell line per tumor type was selected where the derivatives displayed the highest overall levels of cytostatic activity (Table 3). Urea derivative **11j**, the parent structure in the preceding molecular design study, was used as a standard to evaluate the pharmacological model presented herein.

The results presented in Table 3 show clearly that ureas **11b**, **11c**, **11f** and **11j** did not display higher levels of cytostatic activity than doxorubicin. However, the ureas did exhibit promising anticancer activities that exceeded that of amsacrine, especially in the case of the HCT–166, RXF393 and NCI–H522 cancer cell lines (Table 3, Table S2). Against the selected cancer cell lines, urea derivatives **11b**, **11c**, **11f** and **11j** also displayed superior levels of biological activity than both fluorouracil and cisplatin.

In order to provide an overall view of the cytostatic activity of the urea derivatives, the GI_50_ values of each derivative for all cancer cell lines are listed in a decimal logarithmic form in the supplementary materials (Tables S1–S4). In summary, ureas **11b**, **11c** and **11f** were found to be the most cytostatic against leukemia cell lines and moderate activity was also shown against colon cell lines (Table 4, Table S2).

**Figure 6:**
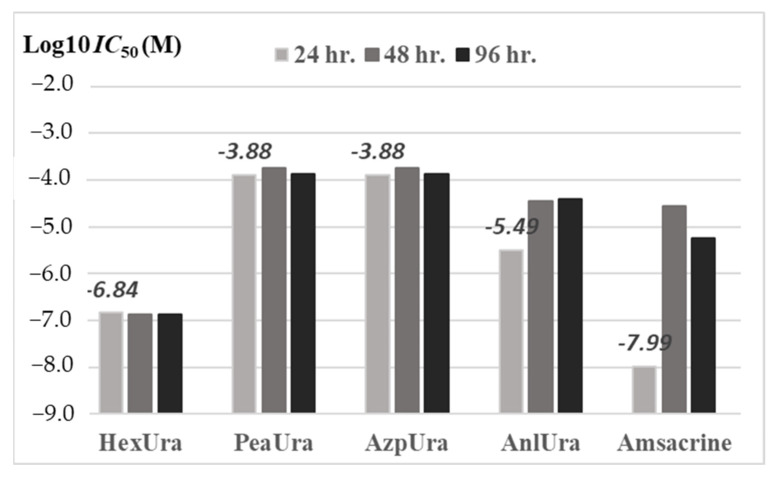
Decimal logarithm of the inhibition concentration (IC_50_) of ureas **11b**, **11c**, **11f**, **11j** and amsacrine against immortalised foreskin fibroblasts (BJ-5ta) after 24, 48 and 96 h determined by real-time cell analysis and dynamic monitoring of cell proliferation. IC_50_ values are given as a molar concentration.

In order to determine the toxicity (IC_50_) of the novel derivatives, the biological activity of ureas **11b**, **11c**, **11f** and **11j** were performed against immortalized foreskin fibroblasts (Table 5). The IC_50_ values obtained in the studies show that azepyl **11b** and phenylethyl **11f** urea exhibited levels of cytotoxicity three orders of magnitude lower than their cytostatic activity. The highest level of cytotoxicity was displayed by hexyl urea **11j**, a level even greater than that of amsacrine after 48 h and 96 h, whereas phenyl urea **11c** was found to be more cytostatic. Figure 6 expresses the IC_50_ values in a decimal logarithm scale in order to allow a better comparison of the cytotoxicity of the proflavine ureas and amsacrine.

## 3. Materials and Methods

### 3.1. General Remarks

All chemicals and reagents were of reagent grade. NMR spectra were measured on Varian VNMRS NMR and Varian Mercury Plus 400 FT NMR spectrometers at room temperature (600, 400 MHz). δ values were referenced on a residual solvent signal as follows: for ^1^H–DMSO-*d_6_* (2.49 ppm) and for ^13^C–DMSO-*d_6_* (39.5 ppm). Reactions were monitored with thin-layer chromatography (TLC) using Silufol plates with detection at 254 nm. Preparative column chromatography was performed using Aluminum oxide Merck 90 neutral (grain size 200 nm). High-resolution mass spectra (HRMS) were recorded on a micrOTOF-QII quadrupole-time-of-flight mass spectrometer (Bruker Daltonics) with an electrospray ionization source. Optical rotations were determined using a P-2000 Jasco polarimeter. Melting points were recorded on a Koffler hot block and are presented uncorrected.

### 3.2. Synthesis

#### 3.2.1. Synthesis of 3,6-Diisothiocyanatoacridine (**9**)

Proflavine hemisulfate hydrate (**7**) (300 mg, 1.43 mmol), thiophogene (0.22 mL, 2.87 mmol) and chloroform (30 mL) were poured into a flask to which 5 mL of the stock solution of sodium carbonate (0.45 g in 25 mL H_2_O) per 5 min was added while the mixture was shaken intensively. A solution of sodium bicarbonate (1.50 g in 15 mL H_2_O) was then added and the reaction mixture was stirred for 20 min. In turn, an organic layer with the soluted product **9** was separated and dried over CaCl_2_. The crude product **9** was purified by flash chromatography on neutral aluminum oxide with chloroform as an eluent. The purified isotiocynate **9** was then crystalized from toluene (50 mL) with the addition of activated coal (100 mg) at 80 °C. All physicochemical properties were in accordance with the previously published data [14].

#### 3.2.2. General Protocol for the Synthesis of Thioureas **10a**–**10k**

Amine (2.0 mmol) was added to a heterogeneous solution of 3,6-diisothiocyanatoacridine (**9**) (60 mg, 0.20 mmol) in methanol (1.50 mL) [14]. The reaction mixture was stirred vigorously and the course of the reaction was monitored using TLC in which chloroform was used as an eluent. When the TLC result was negative in the presence of reactant **9**, diethylether was added and the resultant mixture was stirred for an additional two hours to maximize the precipitation of the product. The heterogeneous mixture was then filtered off and the crude product was washed with ethylacetate then with *n*-hexane in a funnel. The final product was crystalized from the DMF–methanol mixture.

*N,N’-Acridine-3,6-diylbis(N’-cyclohexylthiourea)* (**10a**, 92 mg, 91.3%). Yellow crystalline solid, mp > 200 °C with decomposition. Thiourea was crystalized from the DMSO-diethyleter-methanol mixture. ^1^H NMR (600 MHz, DMSO–*d_6_*) δ 9.80 (s, 2 × NH, 2H) 8.84 (s, H9, 1H) 8.39 (s, H4, H5, 2H), 8.03 (d, *J* = 7.8 Hz, 2 × NH, 2H), 8.00 (d, *J* = 9.1 Hz, H1, H8, 2H), 7.60 (d, *J* = 9.1 Hz, H2, H7, 2H), 4.26–4.05 (m, 2 × NHCH, 2H) 2.08–1.92 (m, 4 × NHCHCH_A_, 4H), 1.84–1.65 (m, 4 × NHCHCH_2_CH_A_, 4H) 1.65–1.52 (m, 2 × NHCHCH_2_CH_2_CH_A_, 2H), 1.47–1.26 (m, 4 × NHCHCH_B_, 4H), 1.47–1.26 (m, 4 × NHCHCH_2_CH_B_, 4H), 1.26–1.11 (m, 2 × NHCHCH_2_CH_2_CH_B_, 2H). ^13^C NMR (150 MHz, DMSO–*d_6_*) δ 179.0 (2 × CS), 149.4 (C4a, C10a), 141.6 (C3, C6), 135.0 (C9), 128.5 (C1, C8), 122.6 (C8a, C9a), 122.5 (C2, C7), 115.6 (C4, C5), 52.3 (2 × NHCH), 31.8 (4 × NHCHCH_2_), 25.2 (2 × NHCHCH_2_CH_2_CH_2_), 24.5 (4 × NHCHCH_2_CH_2_). HRMS (ESI): *m*/*z* calculated for C_27_H_33_N_5_S_2_ [M + H]^+^ 492.22501, found 492.22650.

*N,N’-Acridine-3,6-diylbis(N’-(azepan-1-yl)thiourea)* (**10b**, 96 mg, 95.2%). Yellow crystalline solid, mp > 200 °C with decomposition. Thiourea was crystallized from the DMSO-diethyleter-methanol mixture. ^1^H NMR (400 MHz, DMSO–*d_6_*) δ 9.38 (s, 2 × NH, 2H), 8.88 (s, H9, 1H), 7.97 (s, H1, H8, 2H), 7.85 (d, *J* = 9.1 Hz, H4, H5, 2H), 7.70 (d, *J* = 9.1 Hz, H2, H7, 2H), 4.01–3.80 (m, 4 × NCH_2_, 8H), 1.86–1.77 (m, 4 × NCH_2_CH_2_, 8H), 1.63–1.51 (m, 4 × NCH_2_CH_2_CH_2_, 8H). ^13^C NMR (100 MHz, DMSO–*d_6_*) δ 180.6 (2 × CS), 149.1 (C4a, C10a), 143.2 (C3, C6), 134.8 (C9), 126.9 (C1, C8), 125.9 (C2, C7), 123.3 (C8a, C9a), 119.9 (C4, C5), 50.0 (4 × NCH_2_), 26.9 (4 × NCH_2_CH_2_), 26.3 (2 × NCH_2_CH_2_CH_2_). HRMS (ESI): *m*/*z* calculated for C_27_H_33_N_5_S_2_ [M + H]^+^ 492.22501, found 492.22750.

*N,N’-Acridine-3,6-diylbis(N’-phenylthiourea)* (**10c**, 92 mg, 93.5%). Yellow crystalline solid, mp > 200°C with decomposition. Thiourea was crystalized from the DMSO-diethyleter-methanol mixture. ^1^H NMR (600 MHz, DMSO–*d_6_*) δ 10.28 (bs, 2 × NH, 2H), 10.15 (bs, 2 × NH, 2H), 8.91 (s, H9, 1H), 8.28 (s, H4, H5, 2H), 8.06 (d, *J* = 9.1 Hz, H1, H8, 2H), 7.72 (d, *J* = 9.1 Hz, H2, H7, 2H), 7.58–7.53 (m, 2 × (H2′, H6′), 4H), 7.41–7.34 (m, 2 × (H3’, H5’), 4H), 7.20–7.13 (m, 2 × H4’, 2H). ^13^C NMR (150 MHz, DMSO–*d_6_*) δ 179.5 (2 × CS), 149.3 (C4a, C10a), 141.5 (C3, C6), 139.3 (2 × C1’), 135.2 (C9), 128.6 (2 × (C3’, C5’), 128.4 (C1, C8), 124.7 (2 × C4’), 123.7 (2 × (C2’, C6’)), 123.2 (C2, C7), 123.1 (C8a, C9a), 117.5 (C4, C5). HRMS (ESI): *m*/*z* calculated for C_27_H_21_N_5_S2 [M + H]^+^ 480.13111, found 480.13252.

*N,N’-Acridine-3,6-diylbis(N’-benzylthiourea)* (**10d**, 100 mg, 96.1%). Yellow crystalline solid, mp > 200°C with decomposition. Thiourea was crystalized from the DMSO-diethyleter-methanol mixture. ^1^H NMR (600 MHz, DMSO–*d_6_*) δ 10.08 (s, 2 × NH, 2H), 8.88 (s, H9, 1H), 8.58 (t, *J* = 5.7 Hz, 2 × NH, 2H), 8.35 (s, H4, H5, 2H), 8.04 (d, *J* = 9.1 Hz, H1, H8, 2H), 7.61 (d, *J* = 9.1 Hz, H2, H7, 2H), 7.40 (d, *J* = 7.3 Hz, 2 × (H2’, H6’), 4H), 7.44–7.34 (m, 2 × (H3’, H5’), 4H), 7.32–7.26 (m, 2 × H4’, 2H), 4.81 (d, *J* = 5.7 Hz, 2 × NHCH_2_, 4H). ^13^C NMR (150 MHz, DMSO–*d_6_*) δ 180.6 (CS), 149.4 (C4a, C10a), 141.3 (C3, C6), 138.7 (2 × C1’), 135.1 (C9), 128.7 (C1, C8), 128.4 (2 × (C3’, C5’)), 127.6 (2 × (C2’, C6’)), 127.0 (2 × C4’), 125.9 (C8a, C9a), 122.8 (C2, C7), 116.6 (C4, C5), 47.3 (2 × NHCH_2_). HRMS (ESI): *m*/*z* calculated for C_29_H_25_N_5_S_2_ [M + H]^+^ 508.16241, found 508.16480.

*N,N’-Acridine-3,6-diylbis(N’-(4-methylphenyl)thiourea)* (**10e**, 92 mg, 88.4%). Yellow crystalline solid, mp > 120 °C with decomposition. Thiourea was crystalized from the DMSO-diethyleter-methanol mixture.1H NMR (400 MHz, DMSO–*d_6_*) δ 10.19 (s, 2 × NH, 2H), 10.04 (s, 2 × NH, 2H), 8.89 (s, H9, 1H), 8.26 (s, H4, H5, 2H), 8.04 (d, *J* = 9.1 Hz, H1, H8, 2H), 7.71 (d, *J* = 9.1 Hz, H2, H7, 2H), 7.39 (d, *J* = 7.3 Hz, 2 × (H2’, H6’), 4H), 7.17 (d, *J* = 7.3 Hz, 2 × (H3’, H5’), 4H), 2.29 (s, 2 × CH_3_, 6H). ^13^C NMR (100 MHz, DMSO–*d_6_*) δ 179.7 (2 × CS), 149.4 (C4a, C10a), 141.8 (C3, C6), 136.8 (2 × C1’), 135.4 (2 × C4’), 134.3 (C9), 129.2 (C1, C8), 128.5 (2 × (C3’, C5’)), 124.1 (2 × (C2’, C6’)), 123.4 (C2, C7), 123.2 (C8a, C9a), 117.6 (C4, C5), 20.7 (2 × CH_3_). HRMS (ESI): *m*/*z* calculated for C_29_H_25_N_5_S_2_ [M + H]^+^ 508.16241, found 508.16475.

*N,N’-Acridine-3,6-diylbis(N’-(2-phenylethyl)thiourea)* (**10f**, 100 mg, 91.1%). Yellow crystalline solid, mp > 180 °C with decomposition. Thiourea was crystalized from the DMSO-diethyleter-methanol mixture. ^1^H NMR (400 MHz, DMSO–*d_6_*) δ 10.02 (bs, 2 × NH, 2H), 8.86 (s, H9, 1H), 8.29 (s, H4, H5, 2H), 8.25–8.14 (m, 2 × NH, 2H), 8.01 (d, *J* = 9.1 Hz, H1, H8, 2H), 7.55 (d, *J* = 9.1 Hz, H2, H7, 2H), 7.39–7.28 (m, 2 × (H2’, H6’), 2 × (H3’, H5’), 8H), 7.27–7.21 (m, 2 × H4’, 2H), 3.84–3.74 (m, 2 × NHCH_2_, 4H), 2.95 (t, *J* = 6.4 Hz, 2 × NHCH_2_CH_2_, 4H). ^13^C NMR (100 MHz, DMSO–*d_6_*) δ 180.2 (2 × CS), 149.4 (C4a, C10a), 141.2 (C3, C6), 139.3 (2 × C1’), 135.0 (C9), 128.7 (2 × (C2’, C6’), (C1, C8)), 128.5 (2 × (C3’, C5’), 126.2 (2 × C4’), 122.8 (C8a, C9a), 122.6 (C2, C7), 116.4 (C4, C5), 45.5 (2 × NHCH_2_CH_2_), 34.3 (2 × NHCH_2_CH_2_). HRMS (ESI): *m*/*z* calculated for C_31_H_29_N_5_S_2_ [M + H]^+^ 536.19371, found 536.19362.

*N,N’-Acridine-3,6-diylbis(N’-(2-methylbenzyl)thiourea)* (**10g**, 104 mg, 94.7%). Yellow crystalline solid, mp > 180 °C with decomposition. Thiourea was crystalized from the DMSO-diethyleter-methanol mixture. ^1^H NMR (400 MHz, DMSO–*d_6_*) δ 10.04 (bs, 2 × NH, 2H), 8.87 (s, H9, 1H), 8.60–8.46 (m, 2 × NH, 2H), 8.35 (s, H4, H5, 2H), 8.03 (d, *J* = 9.1 Hz, H1, H8, 2H), 7.61 (d, *J* = 9.1 Hz, H2, H7, 2H), 7.29 (d, *J* = 7.3 Hz, 2 × (H2’, H6’), 4H), 7.18 (d, *J* = 7.3 Hz, 2 × (H3’, H5’), 4H), 4.75 (d, *J* = 5.7 Hz, 2 × NHCH_2_, 4H), 2.30 (s, 2 × CH_3_, 6H). ^13^C NMR (100 MHz, DMSO–*d_6_*) δ 180.5 (2 × CS), 149.4 (C4a, C10a), 141.3 (C3, C6), 136.1 (2 × C1’), 135.5 (2 × C4’), 135.1 (C9), 128.9 (2 × (C3’, C5’), 128.7 (C1, C8), 127.6 (2 × (C2’, C6’), 122.9 (C8a, C9a), 122.8 (C2, C7), 116.5 (C4, C5), 47.0 (2 × NHCH_2_), 20.7 (2 × CH_3_). HRMS (ESI): *m*/*z* calculated for C_31_H_29_N_5_S_2_ [M + H]^+^ 536.19371, found 536.19574.

*N,N’-Acridine-3,6-diylbis(N’-[(1S)-1-phenylethyl]thiourea)* (**10h**, 40 mg, 36.4%). Yellow crystalline solid, mp > 200 °C with decomposition. Thiourea was crystalized from the diethyleter-*n*-hexane mixture. ^1^H NMR (600 MHz, DMSO–*d_6_*) δ 9.89 (s, 2 × NH, 2H), 8.85 (s, H9, 1H), 8.62–8.51 (m, 2 × NH, 2H), 8.41 (s, H4, H5, 2H), 8.01 (d, *J* = 9.1 Hz, H1, H8, 2H), 7.61 (d, *J* = 9.1 Hz, H2, H7, 2H), 7.42 (d, *J* = 7.3 Hz, 2 × (H2’, H6’), 4H), 7.46–7.33 (m, 2 × (H3’, H5’), 4H), 7.31–7.24 (m, 2 × H4’, 2H), 5.67–5.51 (m, 2 × NHCH, 2H), 1.52 (d, *J* = 7.0 Hz, 2 × CH_3_, 6H). ^13^C NMR (150 MHz, DMSO–*d_6_*) δ 179.5 (2 × CS), 149.4 (C4a, C10a), 143.7 (2 × C1’), 141.6 (C3, C6), 135.0 (C9), 128.5 (C1, C8), 128.4 (2 × (C3’, C5’)), 126.9 (2 × C4’), 126.3 (2 × (C2’, C6’)), 122.7 (C8a, C9a), 122.6 (C2, C7), 116.1 (C4, C5), 52.7 (2 × NHCH), 21.9 (2 × CH_3_). [α]*D*_20_ = +33.3 (c 0.06, Methanol). HRMS (ESI): *m*/*z* calculated for C_31_H_29_N_5_S_2_ [M + H]^+^ 536.19371, found 536.19405.

*N,N’-Acridine-3,6-diylbis(N’-[(1R)-1-phenylethyl]thiourea)* (**10i**, 37 mg, 33.7%). Yellow crystalline solid, mp > 200 °C with decomposition. Thiourea was crystalized from the diethyleter-*n*-hexane mixture. ^1^H NMR (400 MHz, DMSO–*d_6_*) δ 9.89 (s, 2 × NH, 2H), 8.85 (s, H9, 1H), 8.62–8.51 (m, 2 × NH, 2H), 8.41 (s, H4, H5, 2H), 8.01 (d, *J* = 9.1 Hz, H1, H8, 2H), 7.61 (d, *J* = 9.1 Hz, H2, H7, 2H), 7.42 (d, *J* = 7.3 Hz, 2 × (H2’, H6’), 4H), 7.46–7.33 (m, 2 × (H3’, H5’), 4H), 7.31–7.24 (m, 2 × H4’, 2H), 5.67–5.51 (m, 2 × NHCH, 2H), 1.52 (d, *J* = 7.0 Hz, 2 × CH_3_, 6H). ^13^C NMR (100 MHz, DMSO–*d_6_*) δ 179.5 (2 × CS), 149.4 (C4a, C10a), 143.7 (2 × C1’), 141.6 (C3, C6), 135.0 (C9), 128.5 (C1, C8), 128.4 (2 × (C3’, C5’)), 126.9 (2 × C4’), 126.3 (2 × (C2’, C6’)), 122.7 (C8a, C9a), 122.6 (C2, C7), 116.1 (C4, C5), 52.7 (2 × NHCH), 21.9 (2 × CH_3_). [α]*D*_20_ = −33.3 (c 0.06, Methanol). HRMS (ESI): *m*/*z* calculated for C_31_H_29_N_5_S_2_ [M + H]^+^ 536.19371, found 536.19574.

*N,N’-Acridine-3,6-diylbis(N’-hexylthiourea)* (**10j**, 90 mg, 90.8%). All physicochemical properties were in accordance with previously published data [14].

#### 3.2.3. General Protocol for the Synthesis of the Ureas **11a**–**11l**

MNO (2.30 mol%) was added to a heterogeneous mixture of urea (50 mg) in methanol (3 mL) [14]. The reaction mixture was stirred vigorously in dark conditions for 3–5 h. The course of the reaction was monitored using TLC with a methanol–ammonium hydroxide mixture at a volume ratio of 1:10 as an eluent. The crude product was then filtered off and washed with methanol (1 mL), diethyleter (1 mL) and ethylacetate (1 mL).

*N,N’-Acridine-3,6-diylbis(N’-cyclohexylurea)* (**11a**, 35 mg, 74.7%). Yellow crystalline solid, mp > 200 °C with decomposition. Thiourea was crystalized from the DMSO-diethyleter-methanol mixture. ^1^H NMR (600 MHz, DMSO–*d_6_*) δ 8.75 (s, 2 × NH, 2H), 8.69 (s, H9, 1H), 8.12 (s, H4, H5, 2H), 7.91 (d, *J* = 9.1 Hz, H1, H8, 2H), 7.43 (d, *J* = 9.1 Hz, H2, H7, 2H), 6.28 (d, *J* = 7.8 Hz, 2 × NH, 2H), 3.60–3.47 (m, 2 × NHCH, 2H), 1.91–1.76 (m, 4 × NHCHCH_A_, 4H), 1.76–1.62 (m, 4 × NHCHCH_2_CH_A_, 4H), 1.62–1.45 (m, 2 × NHCHCH_2_CH_2_CH_A_, 2H), 1.43–1.27 (m, 4 × NHCHCH_2_CH_B_, 4H), 1.27–1.05 (m, 2 × NHCHCH_2_CH_2_CH_B_, 2H), 1.27–1.05 (m, 4 × NHCHCH_B_, 4H). ^13^C NMR (150 MHz, DMSO–*d_6_*) δ 154.2 (2 × CO), 150.1 (C4a, C10a), 142.1 (C3, C6), 134.9 (C9), 129.0 (C1, C8), 121.2 (C8a, C9a), 119.4 (C2, C7), 110.8 (C4, C5), 47.7 (2 × NHCH), 32.9 (4 × NHCHCH_2_), 25.3 (2 × NHCHCH_2_CH_2_CH_2_), 24.4 (4 × NHCHCH_2_CH_2_). HRMS (ESI): *m*/*z* calculated for C_27_H_33_N_5_O_2_ [M + H]^+^ 460.27070, found 460.27032.

*N,N’-Acridine-3,6-diylbis(N’-(azepan-1-yl)urea)* (**11b**, 30 mg, 53.5%). Yellow crystalline solid, mp > 200 °C with decomposition. Thiourea was crystalized from the DMSO-diethyleter-methanol mixture. ^1^H NMR (600 MHz, DMSO–*d_6_*) δ 8.72 (s, H9, 1H), 8.57 (s, 2 × NH, 2H), 8.19 (s, H4, H5, 2H), 7.92 (d, *J* = 9.1 Hz, H1, H8, 2H), 7.71 (d, *J* = 9.1 Hz, H2, H7, 2H), 3.64–3.45 (m, 4 × NCH_2_, 8H), 1.81–1.62 (m, 4 × NCH_2_CH_2_, 8H), 1.62–1.42 (m, 4 × NCH_2_CH_2_CH_2_, 8H). ^13^C NMR (150 MHz, DMSO–*d_6_*) δ 154.8 (2 × CO), 149.8 (C4a, C10a), 142.4 (C3, C6), 134.6 (C9), 128.1 (C1, C8), 121.6 (C8a, C9a), 121.1 (C2, C7), 113.4 (C4, C5), 46.3 (4 × NCH_2_), 28.1 (4 × NCH_2_CH_2_), 26.6 (4 × NCH_2_CH_2_CH_2_). HRMS (ESI): *m*/*z* calculated for C_27_H_33_N_5_O_2_ [M + H]^+^ 460.27070, found 460.27074.

*N,N’-Acridine-3,6-diylbis(N’-phenylurea)* (**11c**, 34 mg, 73.1%). Yellow crystalline solid, mp > 200 °C with decomposition. Thiourea was crystalized from the DMSO-diethyleter-methanol mixture. ^1^H NMR (600 MHz, DMSO–*d_6_*) δ 9.36 (s, 2 × NH, 2H), 9.00 (s, 2 × NH, 2H), 8.89 (s, H9, 1H), 8.31 (s, H4, H5, 2H), 8.05 (d, *J* = 9.1 Hz, H1, H8, 2H), 7.60–7.50 (m, H2, H7, 2 × (H2’, H6’), 6H), 7.40–7.30 (m, 2 × (H3’, H5’), 4H), 7.03–6.97 (m, 2 × H4’, 2H). ^13^C NMR (150 MHz, DMSO–*d_6_*) δ 152.4 (2 × CO), 148.9 (C4a, C10a), 142.3 (C3, C6), 139.4 (2 × C1’), 136.4 (C9), 129.6 (C1, C8), 128.9 (2 × (C3’, C5’)), 122.2 (2 × C4’), 121.5 (C8a, C9a), 119.8 (C2, C7), 118.4 (2 × (C2’, C6’)), 110.6 (C4, C5). HRMS (ESI): *m*/*z* calculated for C_27_H_21_N_5_O_2_ [M + H]^+^ 448.17680, found 448.17961.

*N,N’-Acridine-3,6-diylbis(N’-benzylthiourea)* (**11d**, 30 mg, 64.0%). Yellow crystalline solid, mp > 200 °C with decomposition. Thiourea was crystalized from the DMSO-diethyleter-methanol mixture. ^1^H NMR (600 MHz, DMSO–*d_6_*) δ 9.04 (s, 2 × NH, 2H), 8.72 (s, H9, 1H), 8.17 (s, H4, H5, 2H), 7.93 (d, *J* = 9.1 Hz, H1, H8, 2H), 7.49 (d, *J* = 9.1 Hz, H2, H7, 2H), 7.38–7.31 (m, 2 × (H2’, H6’), 4H), 7.38–7.31 (m, 2 × (H3’, H5’), 4H), 7.30–7.21 (m, 2 × H4’, 2H), 6.84 (t, *J* = 5.7 Hz, 2 × NH, 2H), 4.38 (d, *J* = 5.7 Hz, 2 × NHCH_2_, 4H). ^13^C NMR (150 MHz, DMSO–*d_6_*) δ 155.1 (2 × CO), 150.1 (C4a, C10a), 142.0 (C3, C6), 140.1 (2 × C1’), 134.9 (C9), 129.0 (C1, C8), 128.4 (2 × (C3’, C5’)), 127.2 (2 × (C2’, C6’)), 126.8 (2 × C4’), 121.3 (C8a, C9a), 119.5 (C2, C7), 111.1 (C4, C5), 42.9 (2 × NHCH_2_). HRMS (ESI): *m*/*z* calculated for C_29_H_25_N_5_O_2_ [M + H]^+^ 476.20810, found 476.20822.

*N,N’-Acridine-3,6-diylbis(N’-(4-methylphenyl)urea)* (**11e**, 30 mg, 64.0%). Yellow crystalline solid, mp > 200 °C with decomposition. Thiourea was crystalized from the DMSO-diethyleter-methanol mixture. ^1^H NMR (400 MHz, DMSO–*d_6_*) δ 9.12 (s, 2 × NH, 2H), 8.81–8.76 (m, H9, NH, 2H), 8.24 (s, H4, H5, 2H), 8.00 (d, *J* = 9.1 Hz, H1, H8, 2H), 7.53 (d, *J* = 9.1 Hz, H2, H7, 2H), 7.41 (d, *J* = 7.3 Hz, 2 × (H2’, H6’), 4H), 7.13 (d, *J* = 7.3 Hz, 2 × (H3’, H5’), 4H), 2.26 (s, 2 × CH_3_, 6H). ^13^C NMR (100 MHz, DMSO–*d_6_*) δ 152.5 (2 × CO), 150.0 (C4a, C10a), 141.5 (C3, C6), 136.9 (2 × C1’), 135.5 (C9), 131.0 (2 × C4’), 129.3 (2 × (C3’, C5’)), 129.2 (C1, C8), 121.6 (C8a, C9a), 119.7 (C2, C7), 118.6 (2 × (C2’, C6’)), 111.8 (C4, C5), 20.4 (2 × CH_3_). HRMS (ESI): *m*/*z* calculated for C_29_H_25_N_5_O_2_ [M + H]^+^ 476.20810, found 476.20803.

*N,N’-Acridine-3,6-diylbis(N’-(2-phenylethyl)urea)* (**11f**, 35 mg, 74.5%). Yellow crystalline solid, mp > 200 °C with decomposition. Thiourea was crystalized from the DMSO-diethyleter-methanol mixture. ^1^H NMR (600 MHz, DMSO–*d_6_*) δ 8.95 (s, 2 × NH, 2H), 8.70 (s, H9, 1H), 8.15 (s, H4, H5, 2H), 7.92 (d, *J* = 9.1 Hz, H1, H8, 2H), 7.45 (d, *J* = 9.1 Hz, H2, H7, 2H), 7.37–7.30 (m, 2 × (H3’, H5’), 4H), 7.28 (d, *J* = 7.3 Hz, 2 × (H2’, H6’), 4H), 7.23–7.21 (m, 2 × H4’, 2H), 6.33 (t, *J* = 5.7 Hz, 2 × NH, 2H), 3.42 (m, 2 × NHCH_2_, 4H), 2.81 (t, *J* = 6.4 Hz, 2 × NHCH_2_CH_2_, 4H). ^13^C NMR (150 MHz, DMSO–*d_6_*) δ 155.0 (2 × CO), 150.1 (C4a, C10a), 142.0 (C3, C6), 139.5 (2 × C1’), 134.9 (C9), 129.0 (C1, C8), 128.7 (2 × (C2’, C6’)), 128.4 (2 × (C3’, C5’)), 126.1 (2 × C4’), 121.3 (C8a, C9a), 119.4 (C2, C7), 111.0 (C4, C5), 40.7 (2 × NHCH_2_CH_2_), 35.7 (2 × NHCH_2_CH_2_). HRMS (ESI): *m*/*z* calculated for C_31_H_29_N_5_O_2_ [M + H]^+^ 504.23940, found 504.24187.

*N,N’-Acridine-3,6-diylbis(N’-(2-methylbenzyl)thiourea)* (**11g**, 35 mg, 74.5%). Yellow crystalline solid, mp > 200 °C with decomposition. Thiourea was crystalized from the DMSO-diethyleter-methanol mixture. ^1^H NMR (400 MHz, DMSO–*d_6_*) δ 9.00 (s, 2 × NH, 2H), 8.71 (s, H9, 1H), 8.16 (s, H4, H5, 2H), 7.93 (d, *J* = 9.1 Hz, H1, H8, 2H), 7.48 (d, *J* = 9.1 Hz, H2, H7, 2H), 7.24 (d, *J* = 7.3 Hz, 2 × (H2’, H6’), 4H), 7.16 (t, *J* = 7.5 Hz, 2 × (H3’, H5’), 4H), 6.77 (t, *J* = 5.7 Hz, 2 × NH, 2H), 4.32 (d, *J* = 5.7 Hz, NHCH_2_, 4H), 2.28 (s, 2 × CH_3_, 6H). ^13^C NMR (100 MHz, DMSO–*d_6_*) δ 155.0 (2 × CO), 150.1 (C4a, C10a), 142.0 (C3, C6), 137.0 (2 × C1’), 135.9 (2 × C4’), 135.0 (C9), 129.0 (C1, C8), 128.9 (2 × (C3’, C5’)), 127.2 (2 × (C2’, C6’)), 121.3 (C8a, C9a), 119.5 (C2, C7), 111.1 (C4, C5), 42.6 (2 × NHCH_2_), 20.7 (2 × CH_3_). HRMS (ESI): *m*/*z* calculated for C_31_H_29_N_5_O_2_ [M + H]^+^ 504.23940, found 504.24140.

*N,N’-Acridine-3,6-diylbis(N’-[(1S)-1-phenylethyl]urea)* (**11h**, 30 mg, 63.8%). Yellow crystalline solid, mp > 200 °C with decomposition. Thiourea was crystalized from the diethyleter-*n*-hexane mixture. ^1^H NMR (600 MHz, DMSO–*d_6_*) δ 8.85 (s, 2 × NH, 2H), 8.70 (s, H9, 1H), 8.10 (s, H4, H5, 2H), 7.92 (d, *J* = 9.1 Hz, H1, H8, 2H), 7.44 (d, *J* = 9.1 Hz, H2, H7, 2H), 7.38 (d, *J* = 7.3 Hz, 2 × (H2’, H6’), 4H), 7.36–7.32 (m, 2 × (H3’, H5’), 4H), 7.28–7.21 (m, 2 × H4’, 2H), 6.83 (d, *J* = 5.7 Hz, 2 × NH, 2H), 4.95–4.84 (m, 2 × NHCH, 2H), 1.43 (d, *J* = 7.0 Hz, 2 × CH_3_, 6H). ^13^C NMR (150 MHz, DMSO–*d_6_*) δ 154.2 (2 × CO), 150.0 (C4a, C10a), 145.0 (2 × C1’), 141.9 (C3, C6), 135.0 (C9), 129.1 (C1, C8), 128.4 (2 × (C3’, C5’)), 126.8 (2 × C4’), 125.9 (2 × (C2’, C6’)), 121.3 (C8a, C9a), 119.4 (C2, C7), 110.9 (C4, C5), 48.7 (2 × NHCH), 23.0 (2 × CH_3_). [α]*D*_20_ = +326.6 (c 0.06, DMSO). HRMS (ESI): *m*/*z* calculated for C_31_H_29_N_5_O_2_ [M + H]^+^ 504.23940, found 504.24143.

*N,N’-Acridine-3,6-diylbis(N’-[(1R)-1-phenylethyl]urea)* (**11i**, 35 mg, 74.5%). Yellow crystalline solid, mp > 200 °C with decomposition. Thiourea was crystalized from the diethyleter-*n*-hexane mixture. ^1^H NMR (400 MHz, DMSO–*d_6_*) δ 8.85 (s, 2 × NH, 2H), 8.70 (s, H9, 1H), 8.11 (s, H4, H5, 2H), 7.93 (d, *J* = 9.1 Hz, H1, H8, 2H), 7.44 (d, *J* = 9.1 Hz, H2, H7, 2H), 7.38 (d, *J* = 7.3 Hz, 2 × (H2’, H6’), 4H), 7.36–7.32 (m, 2 × (H3’, H5’), 4H), 7.28–7.21 (m, 2 × H4’, 2H), 6.83 (d, *J* = 5.7 Hz, 2 × NH, 2H), 4.95–4.84 (m, 2 × NHCH, 2H), 1.43 (d, *J* = 7.0 Hz, 2 × CH_3_, 6H). ^13^C NMR (100 MHz, DMSO–*d_6_*) δ 154.2 (2 × CO), 150.0 (C4a, C10a), 145.0 (2 × C1’), 141.9 (C3, C6), 135.0 (C9), 129.1 (C1, C8), 128.4 (2 × (C3’, C5’)), 126.8 (2 × C4’), 125.9 (2 × (C2’, C6’)), 121.3 (C8a, C9a), 119.4 (C2, C7), 110.9 (C4, C5), 48.7 (2 × NHCH), 23.0 (2 × CH_3_). [α]*D*_20_ = −326.6 (c 0.06, DMSO). HRMS (ESI): *m*/*z* calculated for C_31_H_29_N_5_O_2_ [M + H]^+^ 504.23940, found 504.24133.

*N,N’-Acridine-3,6-diylbis(N’-hexylurea)* (**11j**, 35 mg, 31.2%). All physicochemical properties were in accordance with previously published data [14].

### 3.3. Biology

#### 3.3.1. Cell Line

BJ-5ta (ATCC CRL-4001) (immortalized foreskin fibroblasts) were obtained from ATCC and cultured in Dulbecco’s Modified Eagle’s Medium (DMEM) supplemented with M199 medium (4:1), Hygromycin B (0.01 mg/mL) and 10% of FBS (fetal bovine serum) in the presence of 5% CO_2_ in a humidified atmosphere at 37 °C.

#### 3.3.2. Agilent xCELLigence Real-Time Cell Analysis

Fibroblast cells (5 × 10^3^ cells/well) were seeded in 96-well plates (RTCA E-Plates 96) on *xCELLigence* RTCA systems (Agilent). The cells were treated with derivatives **11b**, **11c**, **11f**, **11j** and amsacrine 24 h after seeding. Fibroblast cells were cultured in the absence or presence of tested drugs at concentrations ranging from 100 µM to 100 nM. The cell adhesion and spread without the manipulation of the cells were continuously monitored in 60 min intervals over the course of a 120 h observation period using the *xCELLigence* RTCA system.

##### Statistical Analysis

Experiments under all conditions were performed in at least three independent measurements. The data were analyzed by using the RTCA software Pro 1.2.1 (ACEA Bioscience). Statistical analysis was carried out by a non-parametric method, one-way ANOVA using SigmaPlot (Ver. 12.0). A *p* < 0.05 was considered significant.

#### 3.3.3. Screening of Anticancer Activity-NCI-60 Panels

The anticancer activity of proflavine ureas **11a**–**11j** was tested against NCI-60 panels consisting of sixty human cancer cell lines. Derivatives **11a**–**11j** were initially at a single dose of 10 μM to identify the individual derivatives with the highest level of growth inhibition. Urea derivatives **11b**, **11c**, **11f** and **11j** were found to have exhibited significant growth inhibition in the One-Dose Screen, and these derivatives were further evaluated against NCI-60 panels at five concentration levels to determine the growth inhibition concentration, GI_50_. The GI_50_ values for doxorubicin, fluorouracil and cisplatin were downloaded from the NCI data repository (https://dtp.cancer.gov/databases_tools/data_search.htm).

The screening protocol is described on the web page: https://dtp.cancer.gov/discovery_development/nci-60/methodology.htm.

### 3.4. Molecular Modeling

#### 3.4.1. Normal Mode Analysis

To build urea models I–XXIII, 3D Viewer (ver. 11.01) and ChemSketch (ver. 11.02) software as components of the ACD Labs software package were used [16]. Molecular design studies were performed with Gabedit [19] and MOPAC2016 software using the PM7 method [17]. The optimization of models I–XXIII was controlled by the commands: LET; DDMIN = 0; GNORM = 0.1; AUX; EPS = 78; EF; XYZ. The post-processing normal modes analysis was controlled using the keywords: FORCE; THERMO; PRECISE; EPS = 78.

#### 3.4.2. Calculation of Δ*S*° Value

The change in the standard entropy, Δ*S*°, of specific urea models was calculated using the value of the standard entropy, *S*°, at a temperature of 300 and 340 degrees of Kelvin. The values of the standard entropy are listed in the Mopac output file.

Δ*S*° = *S*°_340K_ − *S*°_300K_

#### 3.4.3. Calculation of Log*P* Value

Log*P* calculations were performed using the “Calculation of Molecular Properties and Prediction of Bioactivity” module within the options of the Molinspiration web tool in order to obtain the log*P* value of individual urea models [18].

#### 3.4.4. Calculation of Score Value

The score value of individual urea models was obtained as the sum of the Log*P* value and the Δ*S*° value.

Score value = Log*P* + Δ*S*°

## 4. Conclusions

Building on our previous studies on the urea derivatives **11j**–**11n**, the pharmacological model was developed in order to facilitate the design of novel urea-based proflavine intercalators which could act as cytostatic substances. The model using a scoring system that utilized the lipophilicity and the change in the standard entropy for individual structures. The physicochemical values of log*P* and Δ*S*° were obtained through a theoretical approach. In order for a given urea model to be synthesized for in vitro screening, it was necessary to achieve a score value which fell within the score range defined by the structure–activity relationship for ureas **11j**–**11n**. Using this scoring system, urea models VIII–XIV, XVII and XVIII were selected as the leading models for synthesis from which all had at least six carbon atoms in their chains.

An improved protocol was applied in the preparation of the urea derivatives in which the yield of the synthesis of 3,6-diisotiocyanatoacridine (**9**) was enhanced substantially, from the previous yield of 53% to 83%.

Urea derivatives **11a**–**11j** were subjected to further investigation through in vitro screening against NCI-60 human cancer cell lines in order to determine their anticancer activity. The strongest cytostatic effect was exhibited by azepyl **11b**, phenyl **11c** and phenylethyl urea **11f**, all of which inhibited the growth of cancer cell lines even at submicromolar concentrations, while ureas **11a**, **11d**, **11e**, **11g**, **11h** and **11i** displayed no effect whatsoever. In comparison to hexyl urea **11j**, the parent structure in the preceding molecular design study, the cytostatic activity of ureas **11b**, **11c** and **11f** was found to have improved only slightly, but this moderate improvement is in line with the results of the scoring system based on opposing physicochemical properties. In contrast, the cytotoxicity of ureas **11b**, **11c** and **11f** was more than three orders of magnitude lower than the cytotoxicity of urea **11j**. In summary, this is the main outcome of the study, especially compared to hexylurea **11j**, which showed almost no selectivity for tumor cells over non-cancer cells.

In conclusion, a series of novel proflavine ureas, derivatives **11a**–**11i**, were synthesized based on molecular modeling design studies. The structure of the novel ureas was proposed on the basis of the pharmacological model. The lipophilicity (Log*P*) and the changes in the standard entropy (Δ*S*°), as the input parameters of the pharmacological model, were obtained using quantum mechanics and cheminformatics. The pharmacological model is based on a scoring system, in which urea models are required to achieve within a specific range in order to be considered suitable for synthesis. The anticancer activity of the synthesized derivatives was evaluated against NCI-60 human cancer cell lines. The strongest anticancer activity was exhibited by ureas azepyl **11b**, phenyl **11c** and phenylethyl **11f**, although the levels of activity were only a slight improvement on those recorded for hexyl urea **11j**. The GI_50_ values recorded for the derivatives against HCT-116 cancer cell line give an indication of the cytostatic effect of the ligands azepyl **11b**–0.44 μM, phenyl **11c**–0.23 μM, phenylethyl **11f**–0.35 μM and hexyl **11j**–0.36 μM. In contrast, the novel ureas **11b**, **11c** and **11f** exhibited levels of cytotoxicity three orders of magnitude lower than that of hexyl urea **11j**.

## Figures and Tables

**Table 1:** Physicochemical and biological data of urea derivatives **11j**–**11n**.

R– ^a^	Urea ^a^	*S*°_300K_^b^	*S*°_340K_^c^	Δ*S*° ^d^	Log*P* ^e^	IC_50_ ^f^	*K*_B_ ^g^
		(Cal/K.Mol)	(Cal/K.Mol)	(Cal/K.Mol)		(μM)	(10^5^)
n-Hexyl	11j	235.004	252.682	17.67	7.03	3.7	0.9
n-Pentyl	11k	215.063	231.541	16.47	6.02	13.3	1.7
n-Butyl	11l	197.441	212.554	15.11	5.01	11.5	2.4
n-Propyl	11m	188.083	201.859	13.77	3.89	29.2	3.8
Ethyl	11n	172.036	184.490	12.45	2.88	60.1	4.2

^a^: A substituent at the location of 3 and 6 of ureas **11j**–**11n**. ^b^: Standard entropy for urea models **11j**–**11n** at a temperature of 300 K. ^c^: Standard entropy for urea models **11j**–**11n** at a temperature of 340 K. ^d^: Gap between the standard entropy for urea models Δ*S*° = *S*°_340K_ − *S*°_300K_. ^e^: Octanol–water partition coefficient as predicted by the web server molinspiration.com. ^f^: Value of the constant IC_50_ determined against a HeLa cancer cell line. ^g^: Value of the binding constant *K*_B_ for the complex of *ct*DNA and ureas **11j**–**11n**.

**Table 2:** Physicochemical data for urea models I–XXIII.

R– ^a^	Model ^a^	*S*°_300K_ ^b^	*S*°_340K_ ^c^	Δ*S*° ^d^	Log*P* ^e^	Score ^f^
		(Cal/K.Mol)	(Cal/K.Mol)	(Cal/K.Mol)		
Aziridine	I	153.9	165.7	11.4	2.35	13.75
*N*,*N’*-Dimethylamine	XXI	171.9	184.5	12.5	1.84	14.34
Azetidine	III	163.9	176.4	12.6	2.89	15.49
Cyklopropylamine	II	176.9	189.8	12.9	2.86	15.76
Pyrolidine	V	184.1	197.8	13.7	3.43	17.13
Cyklobutylamine	IV	186.1	200.1	14	3.45	17.45
*N*,*N’*-Diethylamine	XXII	196.6	211.9	15.1	4.13	19.23
Piperidine	VII	184.6	199.4	14.8	4.44	19.24
Cyklopentylamine	VI	195.6	210.7	15.1	4.93	20.03
Aniline	X	177.7	192.2	14.5	5.53	20.03
Benzylamine	XII	178.1	193.3	15.3	4.93	20.23
Azepane	IX	194.6	210.6	16	5.45	21.45
Cyklohexylamine	VIII	210.2	226.4	16.3	5.94	22.24
4-Methylbenzylamine	XIV	240.4	223.0	17.4	5.38	22.78
4-Methylaniline	XI	205.5	221.8	16.36	6.42	22.78
Phenylethylamine	XIII	211.6	228.8	17.2	5.74	22.94
(*R*/*S*)-Methylethylamine	XVII/XVIII	227.6	245.2	17.6	6.05	23.65
*N*,*N’*-Dipropylamine	XXIII	239.3	257.2	17.9	6.14	24.04
Phenylpropylamine	XV	230.2	248.6	18.48	6.78	25.26
Phenylbutylamine	XVI	235.5	255.2	19.73	7.32	27.05
Adamantylamine	XX	210.2	229.3	19.2	7.98	27.18
*n*-Dekylamine	XIX	283.5	306.3	22.8	11.07	33.87

^a^: A substituent at the location of 3 and 6 in the structure of the ureas models I–XXIII. ^b^: Standard entropy for urea models I–XXIII at a temperature of 300 K. ^c^: Standard entropy for urea models I–XXIII at a temperature of 340 K. ^d^: Gap of the standard entropy for urea models Δ*S*° = *S*°_340K_ − *S*°_300K_. ^e^: Octanol–water partition coefficient predicted by the molinspiration.com web server. ^f^: Score value = Δ*S*° + Log*P.*

**Table 3:** Growth inhibition concentration (GI_50_) of ureas **11b**, **11c**, **11f** and **11j**, fluorouracil, cisplatin, amsacrine and doxorubicin against various human cancer cell lines. GI_50_ values are given as a micromolar concentration.

	HL-60	NCI-H522	HCT-116	U251	LOXIMVI	OVCAR-8	RXF393	DU-145	HS578T
	GI_50_ (μM)								
Hexyl-**11j**	0.26	0.32	0.36	0.65	0.22	0.45	1.41	2.00	0.63
Azepyl-**11b**	0.23	1.58	0.44	0.54	1.41	1.70	0.22	1.74	0.78
Phenyl-**11c**	1.20	1.58	0.23	0.98	0.47	1.15	1.48	2.40	1.51
Phenylethyl-**11f**	0.26	0.32	0.35	0.74	0.20	0.81	1.32	1.23	0.28
Fluorouracil	4.57	11.48	0.49	2.24	0.49	2.40	2.88	0.63	14.13
Cisplatin	6.61	9.12	17.78	11.22	6.61	28.18	20.42	6.76	23.44
Amsacrine	0.02	0.89	0.50	0.21	0.15	0.83	2.24	nd *	nd *
Doxorubicin	0.08	0.03	0.03	0.03	0.03	0.07	0.08	0.07	0.13

* nd—nondetected

**Table 4:** Decimal logarithm of growth inhibition concentration (GI_50_) for ureas **11b**, **11c**, **11f** and **11j**. GI_50_ values are given as a molar concentration.

Cancer Cell Line	Hexyl-11j	Azepyl-11b	Phenyl-11c	Phenylethyl-11f
	Log10 GI_50_ (M)			
CCRF-CEM	−6.56	−6.44	−6.56	−6.52
HL-60(TB)	−6.59	−6.64	−5.92	−6.58
K-562	−6.49	−6.46	−6.44	−6.41
MOLT-4	−6.67	−6.28	−6.25	−6.59
RPMI-8226	−5.79	−6.5	−5.27	−5.65
SR	−6.58	−6.53	−6.54	−6.41
COLO205	−5.85	−5.87	−6.24	−6.5
HCC-2998	−5.84	−5.73	−6.25	−6.35
HCT-116	−6.44	−6.36	−6.63	−6.46
HCT-15	−4.00	−5.94	−4.43	−4.00
HT29	−5.65	−6.37	−5.72	−5.86
KM12	−5.71	−5.72	−5.83	−5.79
SW-620	−6.25	−5.97	−6.48	−6.46
**Average**	−**6.03**	−**6.22**	−**6.04**	−**6.12**

**Table 5:** Inhibition concentration (IC_50_) of ureas **11b**, **11c**, **11f**, **11j** and amsacrine against immortalised foreskin fibroblasts (BJ-5ta) after 24, 48 and 96 h, determined by real-time cell analysis and dynamic monitoring of cell proliferation. IC_50_ values are given as a micromolar concentration.

Urea	IC_50_ (μM)		
	24 h	48 h	96 h
Hexyl-**11j**	0.15	0.13	0.13
Phenylethyl-**11f**	131.00	180.00	137.00
Azepyl-**11b**	131.00	180.00	137.00
Phenyl-**11c**	3.20	34.80	38.50
Amsacrine	0.01	27.70	5.80

## Data Availability

The data presented in this study are available in the article and Supplementary Materials. The GI_50_ values for doxorubicin, fluorouracil, cisplatin, ureas **11b**, **11c**, **11f** and **11j** were downloaded from the NCI data repository (https://dtp.cancer.gov/databases_tools/data_search.htm; https://dtp.cancer.gov/dtpstandard/dwindex/index.jsp). NCS compound identifier: 119875—cisplatin; 19893—fluouracil; 249992—amsacrine; 759155—doxorubicine; 804281—**11b** (azepyl); 804282—**11b** (phenyl); 804286—**11f** (phenylethyl); 804279—**11j** (*n*-hexyl).

## Supplementary Materials

The following are available online. Tables S1–S4; Figures S1 and S2 and ^1^H, ^13^C NMR spectra of the synthetized compounds.
