# Supplementary material for: Synthesis of Novel Biologically Active Proflavine Ureas Designed on the Basis of Predicted Entropy Changes
Source: Molecules. 2021 Aug 11;26(16):4860. doi: 10.3390/molecules26164860 (PMC8398074; doi:10.3390/molecules26164860)
Supplement: Supplementary file 1 [file molecules-26-04860-s001.zip › molecules-1336076-supplementary.pdf]

SUPPLEMENTARY MATERIAL

# Synthesis of novel biologically active proflavine ureas designed on the basis of predicted entropy changes

**Ladislav Janovec[1,*], Eva Kovacova [1], Martina Semelakova[2], Monika Kvakova[3], Daniel Kupka[4] , David Jager[4] and Maria Kozurkova[5,6]**

[1] Department of Organic Chemistry, Faculty of Science, P. J. Safarik University in Kosice, Moyzesova 11, 040 01, Košice, Slovak Republic; ladislav.janovec@upjs.sk (L.J.); eva.kovacovac@student.upjs.sk (E.K.)
[2] Faculty of Medicine, P. J. Safarik University in Košice, Trieda SNP1, 040 11 Kosice, Slovak Republic; martina.semelakova@upjs.sk (M.S.)
[3] Department of Experimental Medicine, Faculty of Medicine, P. J. Safarik University in Kosice, Trieda SNP1, 040 11 Košice, Slovak Republic; monika.kvakova@upjs.sk (M.Kv.)
[4] Institute of Geotechnics, Slovak Academy of Sciences, Watsonova 45, 040 01, Kosice, Slovak Republic; dankup@saske.sk (D.K.); jager@saske.sk (D.J.)
[5] Department of Biochemistry, Faculty of Science, P. J. Safarik University, Moyzesova 11, 040 01 Kosice, Slovak Republic; maria.kozurkova@upjs.sk (M.K.)
[6] Biomedical Research Center, University Hospital Hradec Kralove, Sokolovska 581, 500 05 Hradec Kralove, Czech Republic
* Correspondence: ladislav.janovec@upjs.sk

Table of contents


| | | |
|---|---|---|
| Table S1. | Heat map of the growth inhibition concentration ($GI_{50}$ [M]) in a decimal logarithm for ureas hexyl **11j**, azepyl **11b**, phenyl **11c** and phenylethyl **11f** and doxorubicine against NCI human cancer cell lines. | p. 2 |
| Table S2. | Heat map of the growth inhibition concentration ($GI_{50}$ [M]) in a decimal logarithm for ureas hexyl **11j**, azepyl **11b**, phenyl **11c** and phenylethyl **11f** and amsacrine against NCI human cancer cell lines. | p. 4 |
| Table S3. | Heat map of the growth inhibition concentration ($GI_{50}$ [M]) in a decimal logarithm for ureas hexyl **11j**, azepyl **11b**, phenyl **11c** and phenylethyl **11f** and cispalatin against NCI human cancer cell lines. | p. 6 |
| Table S4. | Heat map of the growth inhibition concentration ($GI_{50}$ [M]) in a decimal logarithm for ureas hexyl **11j**, azepyl **11b**, phenyl **11c** and phenylethyl **11f** and fluorouracil against NCI human cancer cell lines. | p. 8 |
| Figure S1. | The effect of different compounds on fibroblast cells in the RTCA system. The cytotoxic activity is presented by **11b**, **11f**, **11c**, **11j**, amsacrine at concentrations of 25 nM. Measured electrode impedance is expressed as Slope (proliferation rate, 1/hr). | p. 10 |
| Figure S2. | The effect of different compounds on fibroblast cells in the RTCA system. The cytotoxic activity is presented by **11b**, **11f**, **11c**, **11j**, amsacrine at concentrations of 50 nM. Measured electrode impedance is expressed as Slope (proliferation rate, 1/hr). | p. 10 |
| NMR spectra | $^{1}H$ and $^{13}C$ NMR data for the thioureas **10a** – **10i** | p. 11 |
| NMR spectra | $^{1}H$ and $^{13}C$ NMR data for the ureas **11a** – **11i** | p. 19 |

**Table S1.** Heat map of the growth inhibition concentration ($GI_{50}$ [M]) in a decimal logarithm for ureas hexyl **11j**, azepyl **11b**, phenyl **11c** and phenylethyl **11f** and doxorubicine against NCI human cancer cell lines.

| | | Doxorubicine | Hexyl Urea 11l | Azepyl Urea 11b | Phenyl Urea 11c | Phen.Ethyl Urea 11f |
|---|---|---|---|---|---|---|
| *Small Cell Lung* | DMS114 | nd | nd | nd | nd | nd |
| *Small Cell Lung* | DMS273 | nd | nd | nd | nd | nd |
| *Leukemia* | CCRF-CEM | -7,73 | -6,56 | -6,44 | -6,56 | -6,52 |
| *Leukemia* | HL-60(TB) | -7,11 | -6,59 | -6,64 | -5,92 | -6,58 |
| *Leukemia* | K-562 | -7,05 | -6,49 | -6,46 | -6,44 | -6,41 |
| *Leukemia* | MOLT-4 | -7,73 | -6,67 | -6,28 | -6,25 | -6,59 |
| *Leukemia* | RPMI-8226 | -7,04 | -5,79 | -6,5 | -5,27 | -5,65 |
| *Leukemia* | SR | -8,25 | -6,58 | -6,53 | -6,54 | -6,41 |
| *Non-SmallCellLung* | A549/ATCC | -7,54 | nd | -5,76 | -4,95 | -5,5 |
| *Non-SmallCellLung* | EKVX | -6,7 | -5,24 | -5,84 | -5,37 | -5,46 |
| *Non-SmallCellLung* | HOP-18 | nd | nd | nd | nd | nd |
| *Non-SmallCellLung* | HOP-62 | -7,48 | -5,71 | -5,86 | -5,8 | -5,81 |
| *Non-SmallCellLung* | HOP-92 | -7,63 | -5,78 | -5,8 | -5,8 | -5,83 |
| *Non-SmallCellLung* | NCI-H226 | -7,7 | -5,31 | -5,75 | -5,77 | -5,71 |
| *Non-SmallCellLung* | NCI-H23 | -7,32 | -5,89 | -5,77 | -5,83 | -6,07 |
| *Non-SmallCellLung* | NCI-H322M | -6,54 | -5,84 | nd | -5,76 | -5,94 |
| *Non-SmallCellLung* | NCI-H460 | -8,25 | -5,98 | -5,7 | -5,4 | -5,9 |
| *Non-SmallCellLung* | NCI-H522 | -7,48 | -6,49 | -5,8 | -5,8 | -6,49 |
| *Non-SmallCellLung* | LXFL529 | nd | nd | nd | nd | nd |
| *Colon* | COLO205 | -7,04 | -5,85 | -5,87 | -6,24 | -6,5 |
| *Colon* | DLD-1 | nd | nd | nd | nd | nd |
| *Colon* | HCC-2998 | -7,17 | -5,84 | -5,73 | -6,25 | -6,35 |
| *Colon* | HCT-116 | -7,49 | -6,44 | -6,36 | -6,63 | -6,46 |
| *Colon* | HCT-15 | -6,04 | -4 | -5,94 | -4,43 | -4 |
| *Colon* | HT29 | -7,02 | -5,65 | -6,37 | -5,72 | -5,86 |
| *Colon* | KM12 | -6,87 | -5,71 | -5,72 | -5,83 | -5,79 |
| *Colon* | KM20L2 | nd | nd | nd | nd | nd |
| *Colon* | SW-620 | -7,51 | -6,25 | -5,97 | -6,48 | -6,46 |
| *CNS* | SF-268 | -7,21 | -5,98 | -5,78 | -5,78 | -6,48 |
| *CNS* | SF-295 | -7,43 | -5,68 | -6,11 | -5,74 | -5,71 |
| *CNS* | SF-539 | -7,41 | -5,78 | -6,08 | -5,79 | -5,88 |
| *CNS* | SNB-19 | -7,61 | -5,77 | -5,84 | -5,8 | -5,86 |
| *CNS* | SNB-75 | -7,47 | -5,82 | -5,9 | -5,71 | -5,87 |
| *CNS* | SNB-78 | nd | nd | nd | nd | nd |
| *CNS* | U251 | -7,6 | -6,19 | -6,27 | -6,01 | -6,13 |
| *CNS* | XF498 | nd | nd | nd | nd | nd |
| *Melanoma* | LOXIMVI | -7,6 | -6,66 | -5,85 | -6,33 | -6,7 |
| *Melanoma* | MALME-3M | -7,34 | -5,79 | -5,95 | -5,81 | -6,23 |
| *Melanoma* | M14 | -7,07 | -5,66 | -5,88 | -5,78 | -5,88 |
| *Melanoma* | MDA-MB-435 | -6,96 | -6,39 | -5,93 | -5,78 | -6,09 |
| *Melanoma* | M19-MEL | nd | nd | nd | nd | nd |
| *Melanoma* | SK-MEL-2 | -6,85 | -5,72 | -5,79 | -5,76 | -5,86 |
| *Melanoma* | SK-MEL-28 | -7,1 | -5,76 | -5,88 | -5,74 | -5,85 |
| *Melanoma* | SK-MEL-5 | -7,43 | nd | -5,83 | -5,78 | -5,83 |
| *Melanoma* | UACC-257 | -6,99 | nd | -5,74 | -5,79 | -5,73 |
| *Melanoma* | UACC-62 | -7,46 | -5,92 | -5,77 | -5,76 | -5,89 |
| *Ovarian* | IGROV1 | -7,04 | -5,74 | -5,72 | -5,78 | -5,84 |
| *Ovarian* | OVCAR-3 | -6,78 | -5,93 | -5,7 | -5,96 | -5,98 |
| *Ovarian* | OVCAR-4 | -6,88 | -5,7 | -5,73 | -5,95 | -5,83 |
| *Ovarian* | OVCAR-5 | -6,67 | -5,78 | nd | -5,51 | -5,69 |
| *Ovarian* | OVCAR-8 | -7,17 | -6,35 | -5,77 | -5,94 | -6,09 |
| *Ovarian* | NCI/ADR-RES | -4,97 | -4,08 | -5,61 | -4,16 | -4 |
| *Ovarian* | SK-OV-3 | -6,97 | -5,78 | -5,71 | -5,67 | -5,82 |
| *Renal* | 786-0 | -7,49 | -5,6 | -6,46 | -5,79 | -5,68 |
| *Renal* | A498 | -7,32 | -5,73 | -5,69 | -5,56 | -5,87 |
| *Renal* | ACHN | -7,55 | -4,36 | -5,81 | -5,45 | -4,68 |
| *Renal* | CAKI-1 | -6,75 | -4,58 | -5,78 | -5,53 | -5,33 |
| *Renal* | RXF393 | -7,12 | -5,85 | -6,66 | -5,83 | -5,88 |

| | | | | | | |
|---|---|---|---|---|---|---|
| *Renal* | RXF-631 | nd | nd | nd | nd | nd |
| *Renal* | SN12C | -7,6 | -6,18 | -5,77 | -5,87 | -6,38 |
| *Renal* | TK-10 | -6,75 | -4,63 | -5,72 | -5,4 | -5,3 |
| *Renal* | UO-31 | -6,82 | -4 | -5,78 | -4 | -4 |
| *Prostate* | PC-3 | -6,95 | -5,73 | -5,89 | -5,81 | -5,79 |
| *Prostate* | DU-145 | -7,17 | -5,7 | -5,76 | -5,62 | -5,91 |
| *Breast* | MCF7 | -8,03 | -6,49 | -6,19 | -5,96 | -6,54 |
| *Breast* | MDA-MB-231/ATCC | -6,84 | -5,8 | -5,82 | -5,85 | -5,87 |
| *Breast* | HS578T | -6,88 | -6,2 | -6,11 | -5,82 | -6,56 |
| *Breast* | MDA-N | nd | nd | nd | nd | nd |
| *Breast* | BT-549 | -7,23 | -5,57 | -5,93 | -5,73 | -5,78 |
| *Breast* | T-47D | -7,39 | -5,85 | -5,69 | -5,82 | -6,01 |
| *Breast* | MDA-MB-468 | -7,6 | -5,8 | -5,81 | -6,24 | -6,09 |

**Table S2**. Heat map of the growth inhibition concentration ($GI_{50}$ [M]) in a decimal logarithm for ureas hexyl **11j**, azepyl **11b**, phenyl **11c** and phenylethyl **11f** and amsacrine against NCI human cancer cell lines.

| | | Amsacrine | Hexyl Urea 11l | Azepyl Urea 11b | Phenyl Urea 11c | Phen.Ethyl Urea 11f |
|---|---|---|---|---|---|---|
| *Small Cell Lung* | DMS114 | -6,94 | nd | nd | nd | nd |
| *Small Cell Lung* | DMS273 | -7,27 | nd | nd | nd | nd |
| *Leukemia* | CCRF-CEM | -7,4 | -6,56 | -6,44 | -6,56 | -6,52 |
| *Leukemia* | HL-60(TB) | -7,62 | -6,59 | -6,64 | -5,92 | -6,58 |
| *Leukemia* | K-562 | -6,06 | -6,49 | -6,46 | -6,44 | -6,41 |
| *Leukemia* | MOLT-4 | -7,81 | -6,67 | -6,28 | -6,25 | -6,59 |
| *Leukemia* | RPMI-8226 | -6,69 | -5,79 | -6,5 | -5,27 | -5,65 |
| *Leukemia* | SR | -7,8 | -6,58 | -6,53 | -6,54 | -6,41 |
| *Non-SmallCellLung* | A549/ATCC | -7,52 | nd | -5,76 | -4,95 | -5,5 |
| *Non-SmallCellLung* | EKVX | -5,15 | -5,24 | -5,84 | -5,37 | -5,46 |
| *Non-SmallCellLung* | HOP-18 | -6,06 | nd | nd | nd | nd |
| *Non-SmallCellLung* | HOP-62 | -7,09 | -5,71 | -5,86 | -5,8 | -5,81 |
| *Non-SmallCellLung* | HOP-92 | -6,64 | -5,78 | -5,8 | -5,8 | -5,83 |
| *Non-SmallCellLung* | NCI-H226 | -6,87 | -5,31 | -5,75 | -5,77 | -5,71 |
| *Non-SmallCellLung* | NCI-H23 | -6,86 | -5,89 | -5,77 | -5,83 | -6,07 |
| *Non-SmallCellLung* | NCI-H322M | -5,18 | -5,84 | nd | -5,76 | -5,94 |
| *Non-SmallCellLung* | NCI-H460 | -7,88 | -5,98 | -5,7 | -5,4 | -5,9 |
| *Non-SmallCellLung* | NCI-H522 | -6,05 | -6,49 | -5,8 | -5,8 | -6,49 |
| *Non-SmallCellLung* | LXFL529 | -5,27 | nd | nd | nd | nd |
| *Colon* | COLO205 | -5,41 | -5,85 | -5,87 | -6,24 | -6,5 |
| *Colon* | DLD-1 | -5,45 | nd | nd | nd | nd |
| *Colon* | HCC-2998 | -5,73 | -5,84 | -5,73 | -6,25 | -6,35 |
| *Colon* | HCT-116 | -6,3 | -6,44 | -6,36 | -6,63 | -6,46 |
| *Colon* | HCT-15 | -5,94 | -4 | -5,94 | -4,43 | -4 |
| *Colon* | HT29 | -5,79 | -5,65 | -6,37 | -5,72 | -5,86 |
| *Colon* | KM12 | -5,88 | -5,71 | -5,72 | -5,83 | -5,79 |
| *Colon* | KM20L2 | -5,66 | nd | nd | nd | nd |
| *Colon* | SW-620 | -6,72 | -6,25 | -5,97 | -6,48 | -6,46 |
| *CNS* | SF-268 | -6,25 | -5,98 | -5,78 | -5,78 | -6,48 |
| *CNS* | SF-295 | -7,22 | -5,68 | -6,11 | -5,74 | -5,71 |
| *CNS* | SF-539 | -6,66 | -5,78 | -6,08 | -5,79 | -5,88 |
| *CNS* | SNB-19 | -6,51 | -5,77 | -5,84 | -5,8 | -5,86 |
| *CNS* | SNB-75 | -6,38 | -5,82 | -5,9 | -5,71 | -5,87 |
| *CNS* | SNB-78 | -5,24 | nd | nd | nd | nd |
| *CNS* | U251 | -6,68 | -6,19 | -6,27 | -6,01 | -6,13 |
| *CNS* | XF498 | -5,87 | nd | nd | nd | nd |
| *Melanoma* | LOXIMVI | -6,81 | -6,66 | -5,85 | -6,33 | -6,7 |
| *Melanoma* | MALME-3M | -6,21 | -5,79 | -5,95 | -5,81 | -6,23 |
| *Melanoma* | M14 | -6,6 | -5,66 | -5,88 | -5,78 | -5,88 |
| *Melanoma* | MDA-MB-435 | nd | -6,39 | -5,93 | -5,78 | -6,09 |
| *Melanoma* | M19-MEL | -5,81 | nd | nd | nd | nd |
| *Melanoma* | SK-MEL-2 | -5,26 | -5,72 | -5,79 | -5,76 | -5,86 |
| *Melanoma* | SK-MEL-28 | -5,46 | -5,76 | -5,88 | -5,74 | -5,85 |
| *Melanoma* | SK-MEL-5 | -6,48 | nd | -5,83 | -5,78 | -5,83 |
| *Melanoma* | UACC-257 | -5,61 | nd | -5,74 | -5,79 | -5,73 |
| *Melanoma* | UACC-62 | -6,7 | -5,92 | -5,77 | -5,76 | -5,89 |
| *Ovarian* | IGROV1 | -5,38 | -5,74 | -5,72 | -5,78 | -5,84 |
| *Ovarian* | OVCAR-3 | -5,66 | -5,93 | -5,7 | -5,96 | -5,98 |
| *Ovarian* | OVCAR-4 | -5,57 | -5,7 | -5,73 | -5,95 | -5,83 |
| *Ovarian* | OVCAR-5 | -5,8 | -5,78 | nd | -5,51 | -5,69 |
| *Ovarian* | OVCAR-8 | -6,08 | -6,35 | -5,77 | -5,94 | -6,09 |
| *Ovarian* | NCI/ADR-RES | nd | -4,08 | -5,61 | -4,16 | -4 |
| *Ovarian* | SK-OV-3 | -6,42 | -5,78 | -5,71 | -5,67 | -5,82 |
| *Renal* | 786-0 | -6,76 | -5,6 | -6,46 | -5,79 | -5,68 |
| *Renal* | A498 | -6,16 | -5,73 | -5,69 | -5,56 | -5,87 |
| *Renal* | ACHN | -7,43 | -4,36 | -5,81 | -5,45 | -4,68 |
| *Renal* | CAKI-1 | -7,52 | -4,58 | -5,78 | -5,53 | -5,33 |
| *Renal* | RXF393 | -5,65 | -5,85 | -6,66 | -5,83 | -5,88 |

| | | | | | | |
|---|---|---|---|---|---|---|
| *Renal* | RXF-631 | -6,41 | nd | nd | nd | nd |
| *Renal* | SN12C | -7,06 | -6,18 | -5,77 | -5,87 | -6,38 |
| *Renal* | TK-10 | -5,58 | -4,63 | -5,72 | -5,4 | -5,3 |
| *Renal* | UO-31 | -5,96 | -4 | -5,78 | -4 | -4 |
| *Prostate* | PC-3 | nd | -5,73 | -5,89 | -5,81 | -5,79 |
| *Prostate* | DU-145 | nd | -5,7 | -5,76 | -5,62 | -5,91 |
| *Breast* | MCF7 | nd | -6,49 | -6,19 | -5,96 | -6,54 |
| *Breast* | MDA-MB-231/ATCC | nd | -5,8 | -5,82 | -5,85 | -5,87 |
| *Breast* | HS578T | nd | -6,2 | -6,11 | -5,82 | -6,56 |
| *Breast* | MDA-N | nd | nd | nd | nd | nd |
| *Breast* | BT-549 | nd | -5,57 | -5,93 | -5,73 | -5,78 |
| *Breast* | T-47D | nd | -5,85 | -5,69 | -5,82 | -6,01 |
| *Breast* | MDA-MB-468 | nd | -5,8 | -5,81 | -6,24 | -6,09 |

**Table S3.** Heat map of the growth inhibition concentration ($GI_{50}$ [M]) in a decimal logarithm for ureas hexyl **11j**, azepyl **11b**, phenyl **11c** and phenylethyl **11f** and cisplatin against NCI human cancer cell lines.

| | | **Cisplatin** | **Hexyl Urea 11l** | **Azepyl Urea 11b** | **Phenyl Urea 11c** | **Phen.Ethyl Urea 11f** |
|---|---|---|---|---|---|---|
| *Small Cell Lung* | DMS114 | -5,24 | nd | nd | nd | nd |
| *Small Cell Lung* | DMS273 | -5,41 | nd | nd | nd | nd |
| *Leukemia* | CCRF-CEM | -5,26 | -6,56 | -6,44 | -6,56 | -6,52 |
| *Leukemia* | HL-60(TB) | -5,18 | -6,59 | -6,64 | -5,92 | -6,58 |
| *Leukemia* | K-562 | -4,58 | -6,49 | -6,46 | -6,44 | -6,41 |
| *Leukemia* | MOLT-4 | -4,88 | -6,67 | -6,28 | -6,25 | -6,59 |
| *Leukemia* | RPMI-8226 | -4,77 | -5,79 | -6,5 | -5,27 | -5,65 |
| *Leukemia* | SR | -5,11 | -6,58 | -6,53 | -6,54 | -6,41 |
| *Non-SmallCellLung* | A549/ATCC | -4,66 | nd | -5,76 | -4,95 | -5,5 |
| *Non-SmallCellLung* | EKVX | -4,45 | -5,24 | -5,84 | -5,37 | -5,46 |
| *Non-SmallCellLung* | HOP-18 | -4,37 | nd | nd | nd | nd |
| *Non-SmallCellLung* | HOP-62 | -5,06 | -5,71 | -5,86 | -5,8 | -5,81 |
| *Non-SmallCellLung* | HOP-92 | -4,71 | -5,78 | -5,8 | -5,8 | -5,83 |
| *Non-SmallCellLung* | NCI-H226 | -4,59 | -5,31 | -5,75 | -5,77 | -5,71 |
| *Non-SmallCellLung* | NCI-H23 | -5,41 | -5,89 | -5,77 | -5,83 | -6,07 |
| *Non-SmallCellLung* | NCI-H322M | -4,51 | -5,84 | nd | -5,76 | -5,94 |
| *Non-SmallCellLung* | NCI-H460 | -5,63 | -5,98 | -5,7 | -5,4 | -5,9 |
| *Non-SmallCellLung* | NCI-H522 | -5,04 | -6,49 | -5,8 | -5,8 | -6,49 |
| *Non-SmallCellLung* | LXFL529 | -4,4 | nd | nd | nd | nd |
| *Colon* | COLO205 | -4,16 | -5,85 | -5,87 | -6,24 | -6,5 |
| *Colon* | DLD-1 | -4,68 | nd | nd | nd | nd |
| *Colon* | HCC-2998 | -4,75 | -5,84 | -5,73 | -6,25 | -6,35 |
| *Colon* | HCT-116 | -4,75 | -6,44 | -6,36 | -6,63 | -6,46 |
| *Colon* | HCT-15 | -4,41 | -4 | -5,94 | -4,43 | -4 |
| *Colon* | HT29 | -4,4 | -5,65 | -6,37 | -5,72 | -5,86 |
| *Colon* | KM12 | -4,41 | -5,71 | -5,72 | -5,83 | -5,79 |
| *Colon* | KM20L2 | -4,43 | nd | nd | nd | nd |
| *Colon* | SW-620 | -4,71 | -6,25 | -5,97 | -6,48 | -6,46 |
| *CNS* | SF-268 | -5,4 | -5,98 | -5,78 | -5,78 | -6,48 |
| *CNS* | SF-295 | -5,19 | -5,68 | -6,11 | -5,74 | -5,71 |
| *CNS* | SF-539 | -5,29 | -5,78 | -6,08 | -5,79 | -5,88 |
| *CNS* | SNB-19 | -4,75 | -5,77 | -5,84 | -5,8 | -5,86 |
| *CNS* | SNB-75 | -5,06 | -5,82 | -5,9 | -5,71 | -5,87 |
| *CNS* | SNB-78 | -5,54 | nd | nd | nd | nd |
| *CNS* | U251 | -4,95 | -6,19 | -6,27 | -6,01 | -6,13 |
| *CNS* | XF498 | nd | nd | nd | nd | nd |
| *Melanoma* | LOXIMVI | -5,18 | -6,66 | -5,85 | -6,33 | -6,7 |
| *Melanoma* | MALME-3M | -4,91 | -5,79 | -5,95 | -5,81 | -6,23 |
| *Melanoma* | M14 | -4,98 | -5,66 | -5,88 | -5,78 | -5,88 |
| *Melanoma* | MDA-MB-435 | -4,61 | -6,39 | -5,93 | -5,78 | -6,09 |
| *Melanoma* | M19-MEL | -4,86 | nd | nd | nd | nd |
| *Melanoma* | SK-MEL-2 | -4,64 | -5,72 | -5,79 | -5,76 | -5,86 |
| *Melanoma* | SK-MEL-28 | -4,66 | -5,76 | -5,88 | -5,74 | -5,85 |
| *Melanoma* | SK-MEL-5 | -4,96 | nd | -5,83 | -5,78 | -5,83 |
| *Melanoma* | UACC-257 | -4,6 | nd | -5,74 | -5,79 | -5,73 |
| *Melanoma* | UACC-62 | -4,89 | -5,92 | -5,77 | -5,76 | -5,89 |
| *Ovarian* | IGROV1 | -5,03 | -5,74 | -5,72 | -5,78 | -5,84 |
| *Ovarian* | OVCAR-3 | -4,99 | -5,93 | -5,7 | -5,96 | -5,98 |
| *Ovarian* | OVCAR-4 | -4,93 | -5,7 | -5,73 | -5,95 | -5,83 |
| *Ovarian* | OVCAR-5 | -4,8 | -5,78 | nd | -5,51 | -5,69 |
| *Ovarian* | OVCAR-8 | -4,55 | -6,35 | -5,77 | -5,94 | -6,09 |
| *Ovarian* | NCI/ADR-RES | -4,7 | -4,08 | -5,61 | -4,16 | -4 |

| | | | | | | |
|---|---|---|---|---|---|---|
| *Ovarian* | SK-OV-3 | -4,63 | -5,78 | -5,71 | -5,67 | -5,82 |
| *Renal* | 786-0 | -5,07 | -5,6 | -6,46 | -5,79 | -5,68 |
| *Renal* | A498 | -4,28 | -5,73 | -5,69 | -5,56 | -5,87 |
| *Renal* | ACHN | -5,14 | -4,36 | -5,81 | -5,45 | -4,68 |
| *Renal* | CAKI-1 | -5,35 | -4,58 | -5,78 | -5,53 | -5,33 |
| *Renal* | RXF393 | -4,69 | -5,85 | -6,66 | -5,83 | -5,88 |
| *Renal* | RXF-631 | -5,12 | nd | nd | nd | nd |
| *Renal* | SN12C | -4,63 | -6,18 | -5,77 | -5,87 | -6,38 |
| *Renal* | TK-10 | -4,47 | -4,63 | -5,72 | -5,4 | -5,3 |
| *Renal* | UO-31 | -4,79 | -4 | -5,78 | -4 | -4 |
| *Prostate* | PC-3 | -4,61 | -5,73 | -5,89 | -5,81 | -5,79 |
| *Prostate* | DU-145 | -5,17 | -5,7 | -5,76 | -5,62 | -5,91 |
| *Breast* | MCF7 | -4,91 | -6,49 | -6,19 | -5,96 | -6,54 |
| *Breast* | MDA-MB-231/ATCC | -4,12 | -5,8 | -5,82 | -5,85 | -5,87 |
| *Breast* | HS578T | -4,63 | -6,2 | -6,11 | -5,82 | -6,56 |
| *Breast* | MDA-N | -4,91 | nd | nd | nd | nd |
| *Breast* | BT-549 | -4,61 | -5,57 | -5,93 | -5,73 | -5,78 |
| *Breast* | T-47D | -4,3 | -5,85 | -5,69 | -5,82 | -6,01 |
| *Breast* | MDA-MB-468 | -5,2 | -5,8 | -5,81 | -6,24 | -6,09 |

**Table S4.** Heat map of the growth inhibition concentration ($GI_{50}$ [M]) in a decimal logarithm for ureas hexyl **11j**, azepyl **11b**, phenyl **11c** and phenylethyl **11f** and fluorouracil against NCI human cancer cell lines.

| | | Fluoruracil | Hexyl Urea 11l | Azepyl Urea 11b | Phenyl Urea 11c | Phen.Ethyl Urea 11f |
|---|---|---|---|---|---|---|
| *Small Cell Lung* | DMS114 | nd | nd | nd | nd | nd |
| *Small Cell Lung* | DMS273 | nd | nd | nd | nd | nd |
| *Leukemia* | CCRF-CEM | -5,01 | -6,56 | -6,44 | -6,56 | -6,52 |
| *Leukemia* | HL-60(TB) | -5,34 | -6,59 | -6,64 | -5,92 | -6,58 |
| *Leukemia* | K-562 | -5,32 | -6,49 | -6,46 | -6,44 | -6,41 |
| *Leukemia* | MOLT-4 | -6,06 | -6,67 | -6,28 | -6,25 | -6,59 |
| *Leukemia* | RPMI-8226 | -6,89 | -5,79 | -6,5 | -5,27 | -5,65 |
| *Leukemia* | SR | -6,86 | -6,58 | -6,53 | -6,54 | -6,41 |
| *Non-SmallCellLung* | A549/ATCC | -6,45 | nd | -5,76 | -4,95 | -5,5 |
| *Non-SmallCellLung* | EKVX | -4,33 | -5,24 | -5,84 | -5,37 | -5,46 |
| *Non-SmallCellLung* | HOP-18 | nd | nd | nd | nd | nd |
| *Non-SmallCellLung* | HOP-62 | -6,15 | -5,71 | -5,86 | -5,8 | -5,81 |
| *Non-SmallCellLung* | HOP-92 | -4,08 | -5,78 | -5,8 | -5,8 | -5,83 |
| *Non-SmallCellLung* | NCI-H226 | -4,22 | -5,31 | -5,75 | -5,77 | -5,71 |
| *Non-SmallCellLung* | NCI-H23 | -6,2 | -5,89 | -5,77 | -5,83 | -6,07 |
| *Non-SmallCellLung* | NCI-H322M | -6,16 | -5,84 | nd | -5,76 | -5,94 |
| *Non-SmallCellLung* | NCI-H460 | -6,98 | -5,98 | -5,7 | -5,4 | -5,9 |
| *Non-SmallCellLung* | NCI-H522 | -4,94 | -6,49 | -5,8 | -5,8 | -6,49 |
| *Non-SmallCellLung* | LXFL529 | nd | nd | nd | nd | nd |
| *Colon* | COLO205 | -6,52 | -5,85 | -5,87 | -6,24 | -6,5 |
| *Colon* | DLD-1 | nd | nd | nd | nd | nd |
| *Colon* | HCC-2998 | -6,92 | -5,84 | -5,73 | -6,25 | -6,35 |
| *Colon* | HCT-116 | -6,31 | -6,44 | -6,36 | -6,63 | -6,46 |
| *Colon* | HCT-15 | -6,63 | -4 | -5,94 | -4,43 | -4 |
| *Colon* | HT29 | -6,47 | -5,65 | -6,37 | -5,72 | -5,86 |
| *Colon* | KM12 | -6,36 | -5,71 | -5,72 | -5,83 | -5,79 |
| *Colon* | KM20L2 | nd | nd | nd | nd | nd |
| *Colon* | SW-620 | -5,74 | -6,25 | -5,97 | -6,48 | -6,46 |
| *CNS* | SF-268 | -5,53 | -5,98 | -5,78 | -5,78 | -6,48 |
| *CNS* | SF-295 | -6,14 | -5,68 | -6,11 | -5,74 | -5,71 |
| *CNS* | SF-539 | -6,91 | -5,78 | -6,08 | -5,79 | -5,88 |
| *CNS* | SNB-19 | -5,18 | -5,77 | -5,84 | -5,8 | -5,86 |
| *CNS* | SNB-75 | -4,15 | -5,82 | -5,9 | -5,71 | -5,87 |
| *CNS* | SNB-78 | nd | nd | nd | nd | nd |
| *CNS* | U251 | -5,65 | -6,19 | -6,27 | -6,01 | -6,13 |
| *CNS* | XF498 | nd | nd | nd | nd | nd |
| *Melanoma* | LOXIMVI | -6,31 | -6,66 | -5,85 | -6,33 | -6,7 |
| *Melanoma* | MALME-3M | -6,53 | -5,79 | -5,95 | -5,81 | -6,23 |
| *Melanoma* | M14 | -5,74 | -5,66 | -5,88 | -5,78 | -5,88 |
| *Melanoma* | MDA-MB-435 | -6,76 | -6,39 | -5,93 | -5,78 | -6,09 |
| *Melanoma* | M19-MEL | nd | nd | nd | nd | nd |
| *Melanoma* | SK-MEL-2 | -4,18 | -5,72 | -5,79 | -5,76 | -5,86 |
| *Melanoma* | SK-MEL-28 | -5,83 | -5,76 | -5,88 | -5,74 | -5,85 |
| *Melanoma* | SK-MEL-5 | -6,07 | nd | -5,83 | -5,78 | -5,83 |
| *Melanoma* | UACC-257 | -5,26 | nd | -5,74 | -5,79 | -5,73 |
| *Melanoma* | UACC-62 | -6,04 | -5,92 | -5,77 | -5,76 | -5,89 |
| *Ovarian* | IGROV1 | -5,62 | -5,74 | -5,72 | -5,78 | -5,84 |
| *Ovarian* | OVCAR-3 | -7,26 | -5,93 | -5,7 | -5,96 | -5,98 |
| *Ovarian* | OVCAR-4 | -5,01 | -5,7 | -5,73 | -5,95 | -5,83 |
| *Ovarian* | OVCAR-5 | -4,87 | -5,78 | nd | -5,51 | -5,69 |
| *Ovarian* | OVCAR-8 | -5,62 | -6,35 | -5,77 | -5,94 | -6,09 |
| *Ovarian* | NCI/ADR-RES | -6,12 | -4,08 | -5,61 | -4,16 | -4 |
| *Ovarian* | SK-OV-3 | -4,56 | -5,78 | -5,71 | -5,67 | -5,82 |
| *Renal* | 786-0 | -5,93 | -5,6 | -6,46 | -5,79 | -5,68 |
| *Renal* | A498 | -6,18 | -5,73 | -5,69 | -5,56 | -5,87 |
| *Renal* | ACHN | -6,22 | -4,36 | -5,81 | -5,45 | -4,68 |
| *Renal* | CAKI-1 | -6,63 | -4,58 | -5,78 | -5,53 | -5,33 |
| *Renal* | RXF393 | -5,54 | -5,85 | -6,66 | -5,83 | -5,88 |

| | | | | | | |
|---|---|---|---|---|---|---|
| *Renal* | RXF-631 | nd | nd | nd | nd | Nd |
| *Renal* | SN12C | -5,94 | -6,18 | -5,77 | -5,87 | -6,38 |
| *Renal* | TK-10 | -5,56 | -4,63 | -5,72 | -5,4 | -5,3 |
| *Renal* | UO-31 | -5,85 | -4 | -5,78 | -4 | -4 |
| *Prostate* | PC-3 | -5,25 | -5,73 | -5,89 | -5,81 | -5,79 |
| *Prostate* | DU-145 | -6,2 | -5,7 | -5,76 | -5,62 | -5,91 |
| *Breast* | MCF7 | -6,69 | -6,49 | -6,19 | -5,96 | -6,54 |
| *Breast* | MDA-MB-231/ATCC | -4,88 | -5,8 | -5,82 | -5,85 | -5,87 |
| *Breast* | HS578T | -4,85 | -6,2 | -6,11 | -5,82 | -6,56 |
| *Breast* | MDA-N | nd | nd | nd | nd | Nd |
| *Breast* | BT-549 | -5,16 | -5,57 | -5,93 | -5,73 | -5,78 |
| *Breast* | T-47D | -5,01 | -5,85 | -5,69 | -5,82 | -6,01 |
| *Breast* | MDA-MB-468 | -4,05 | -5,8 | -5,81 | -6,24 | -6,09 |

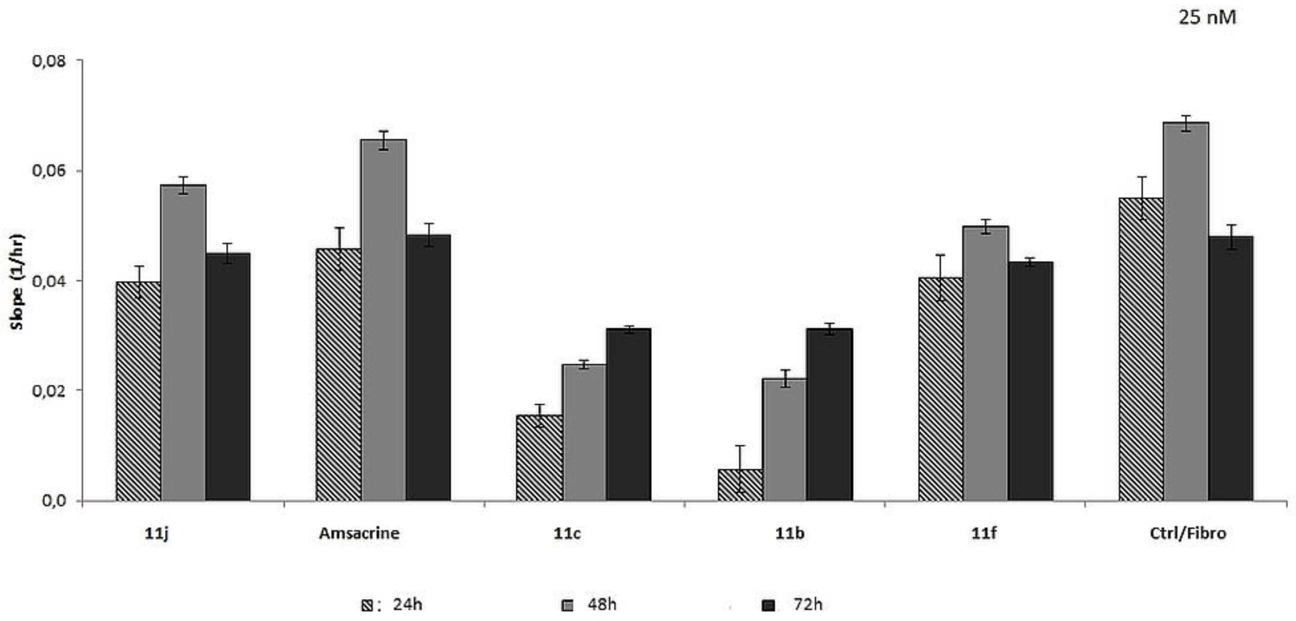


**Figure S1**. The effect of different compounds on fibroblast cells in the RTCA system. The cytotoxic activity is presented by **11b**, **11c**, **11f**, **11j**, amsacrine at concentrations of 25 nM. Measured electrode impedance is expressed as Slope (proliferation rate, 1/hr).

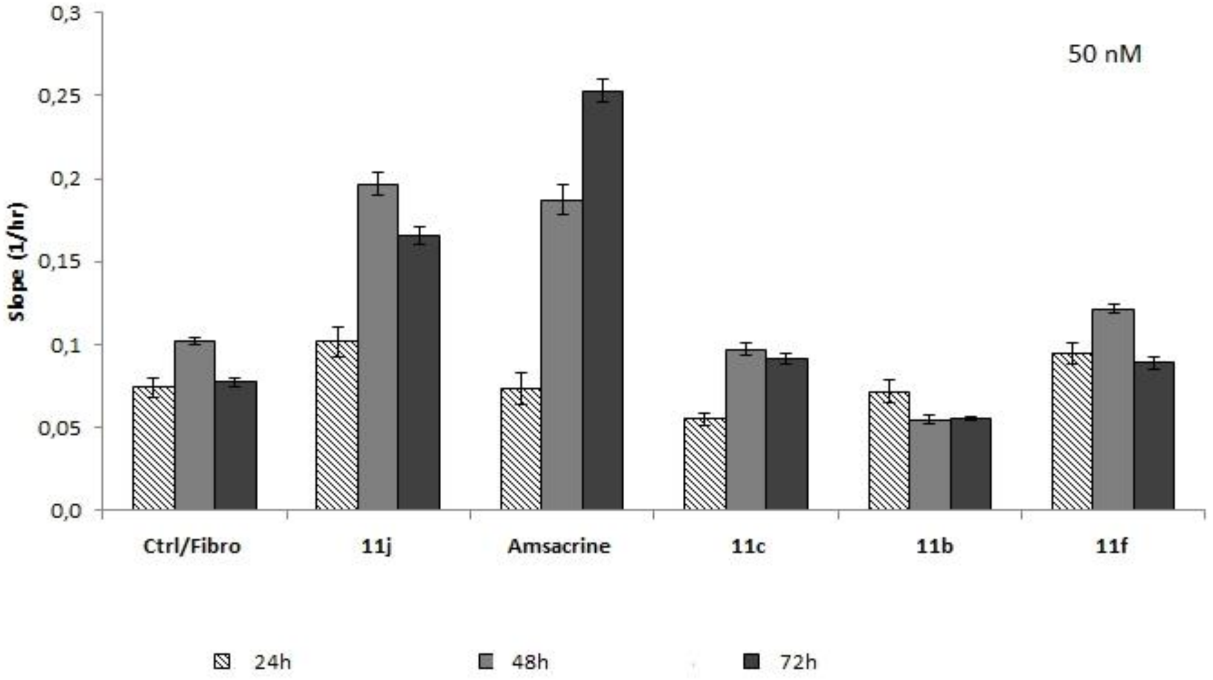


**Figure S2**. The effect of different compounds on fibroblast cells in the RTCA system. The cytotoxic activity is presented by **11b**, **11f**, **11c**, **11j**, amsacrine at concentrations of 50 nM. Measured electrode impedance is expressed as Slope (proliferation rate, 1/hr).

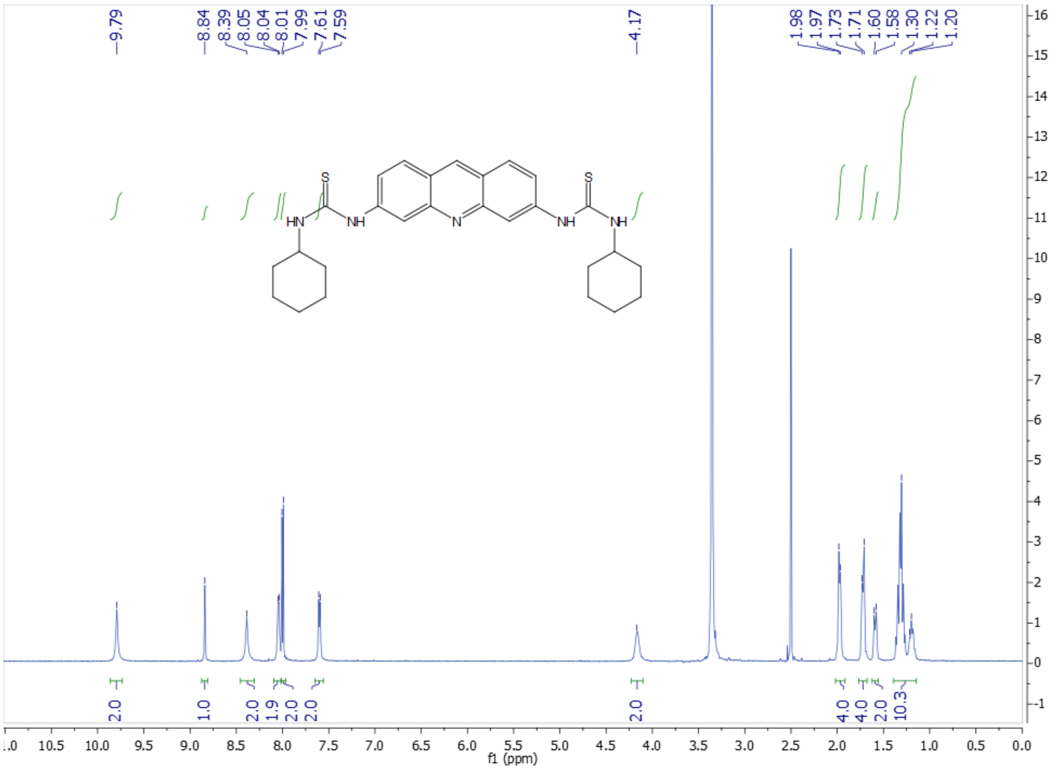

**Figure S3**. $^{1}$H NMR spectra (DMSO-$d_6$, 600 MHz) of the acridine tiourea **10a**.

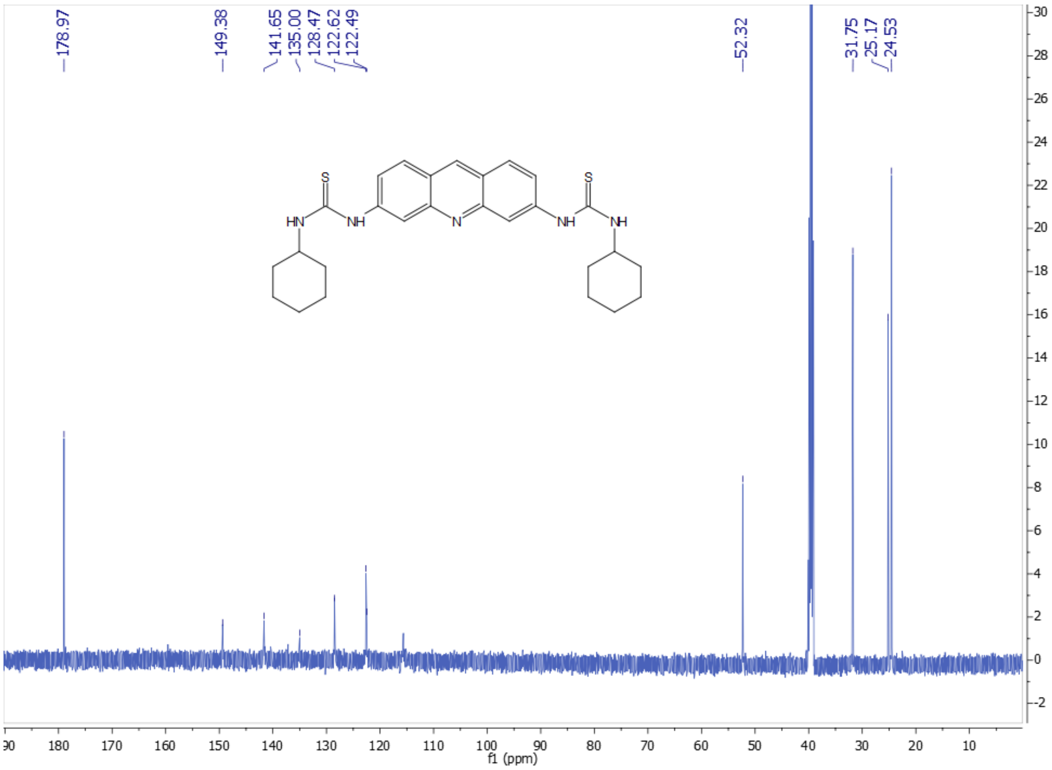

**Figure S4**. $^{13}$C NMR spectra (DMSO-$d_6$, 150 MHz) of the acridine tiourea **10a**.

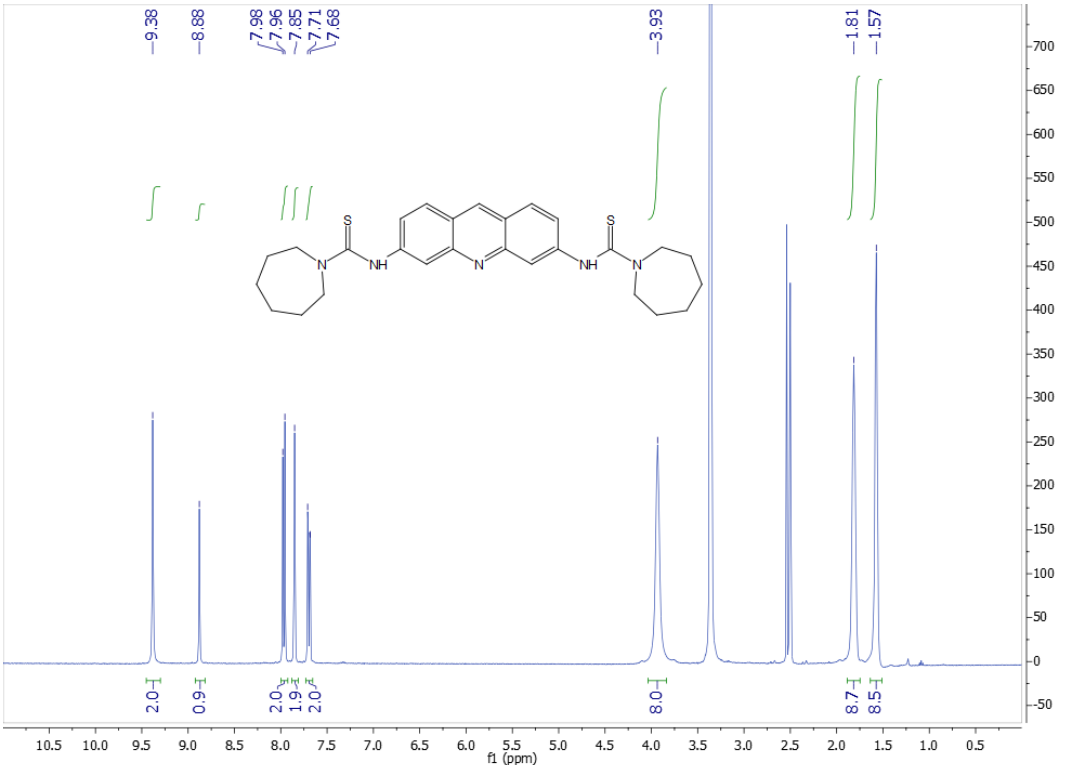


**Figure S5**. $^{1}$H NMR spectra (DMSO-$d_6$, 400 MHz) of the acridine tiourea **10b**.

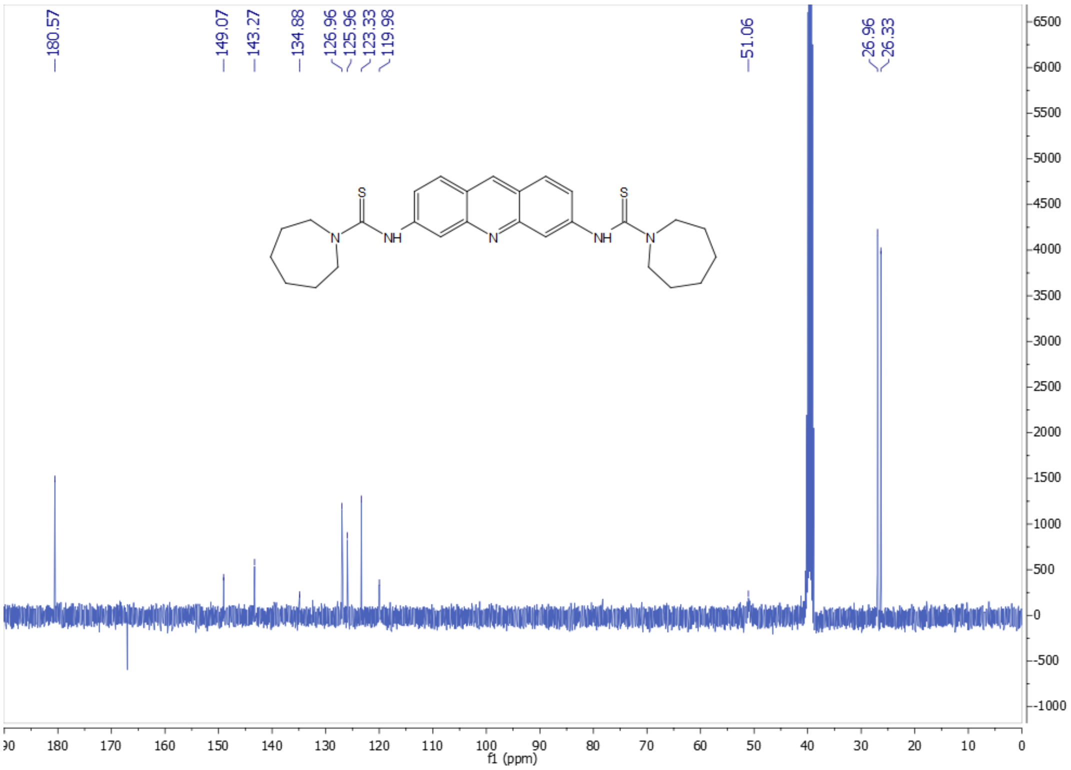


**Figure S6**. $^{13}$C NMR spectra (DMSO-$d_6$, 100 MHz) of the acridine tiourea **10b**.

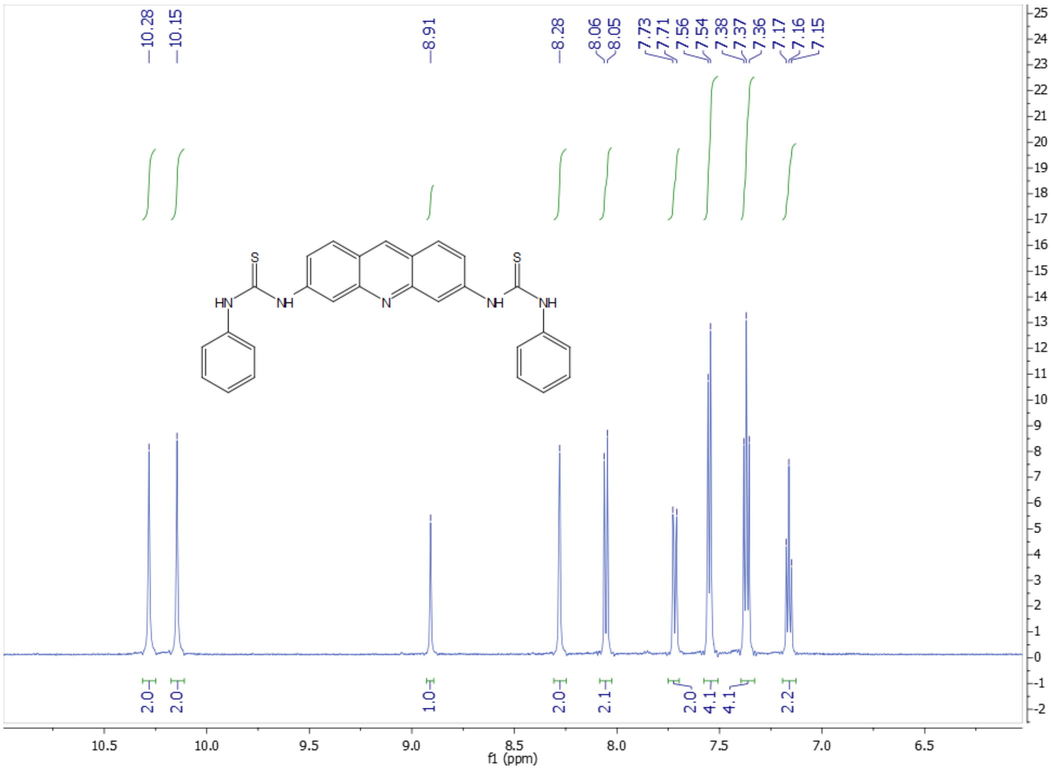


**Figure S7**. $^{1}$H NMR spectra (DMSO-$d_6$, 600 MHz) of the acridine tiourea **10c**.

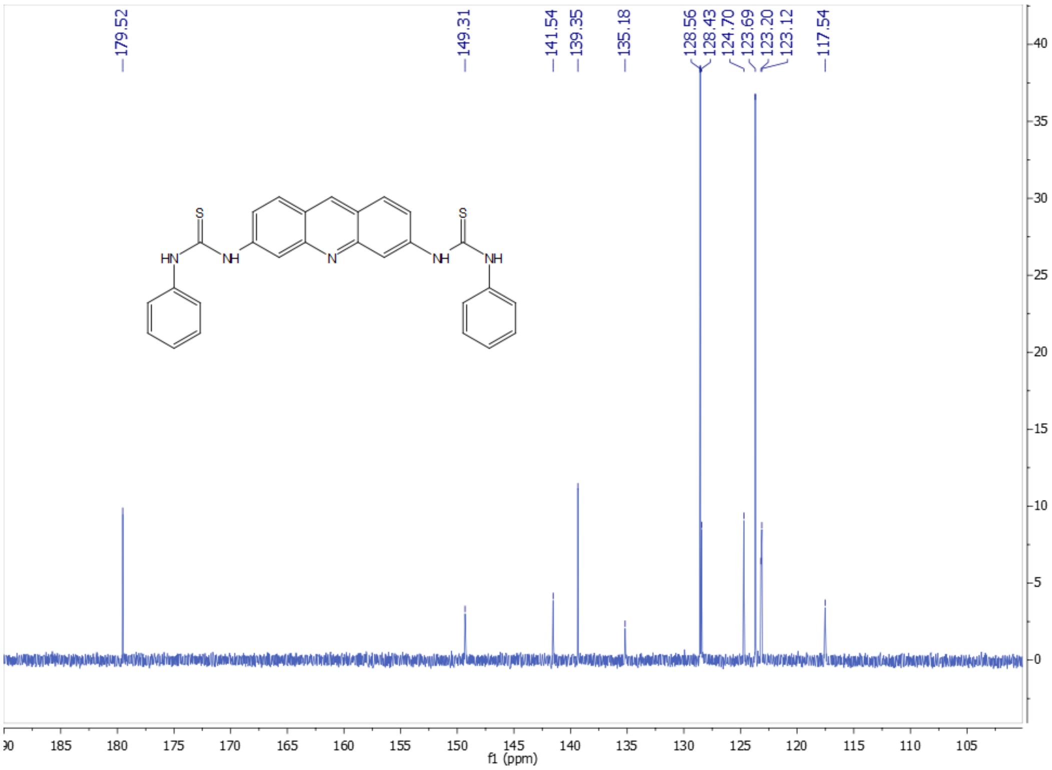


**Figure S8**. $^{13}$C NMR spectra (DMSO-$d_6$, 150 MHz) of the acridine tiourea **10c**.

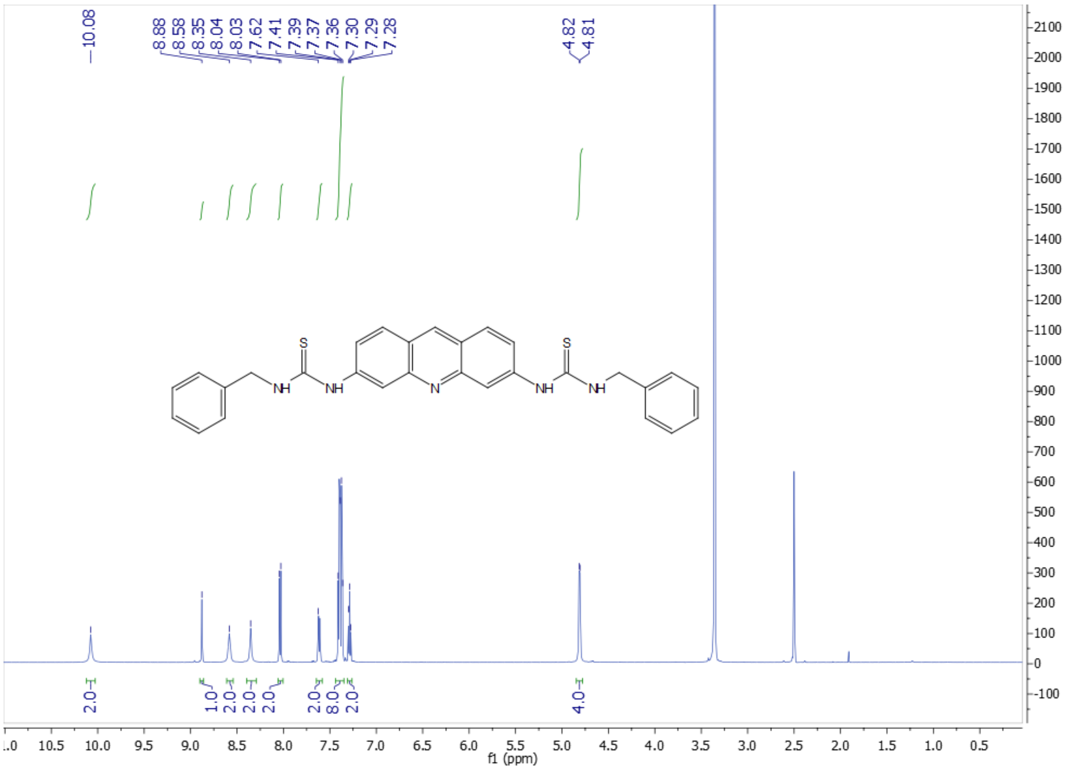

**Figure S9**. $^{1}$H NMR spectra (DMSO-$d_6$, 600 MHz) of the acridine tiourea **10d**.

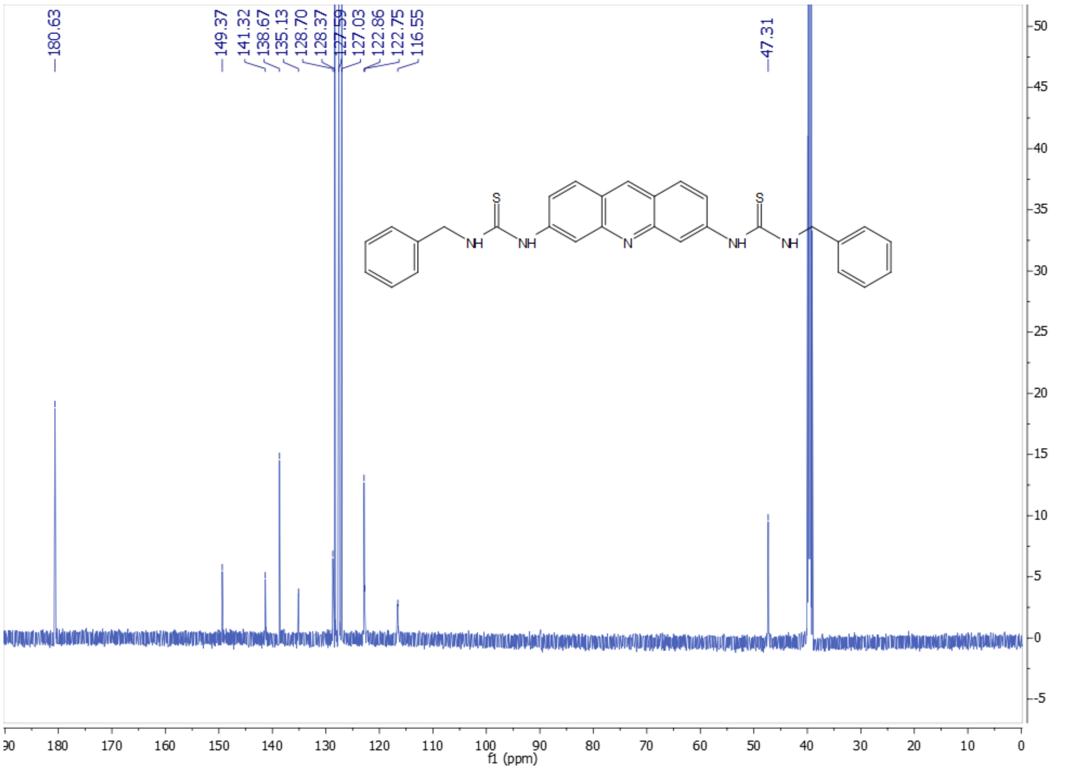

**Figure S10**. $^{13}$C NMR spectra (DMSO-$d_6$, 150 MHz) of the acridine tiourea **10d**.

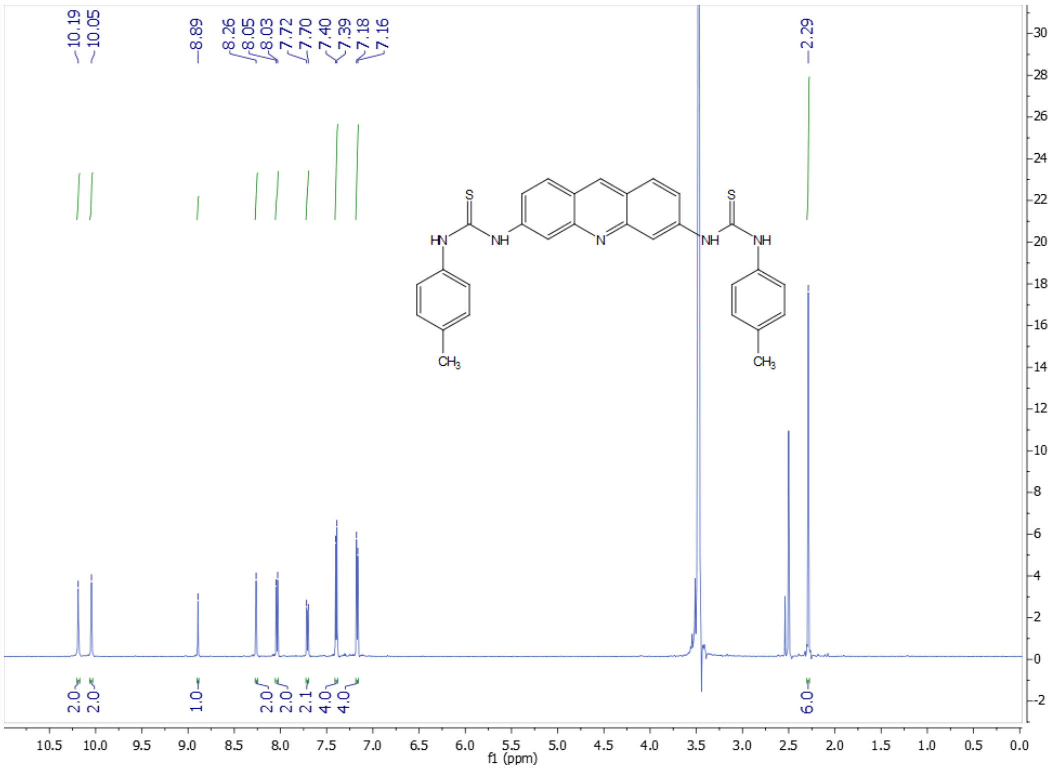

**Figure S11**. $^{1}H$ NMR spectra (DMSO-$d_6$, 600 MHz) of the acridine tiourea **10e**.

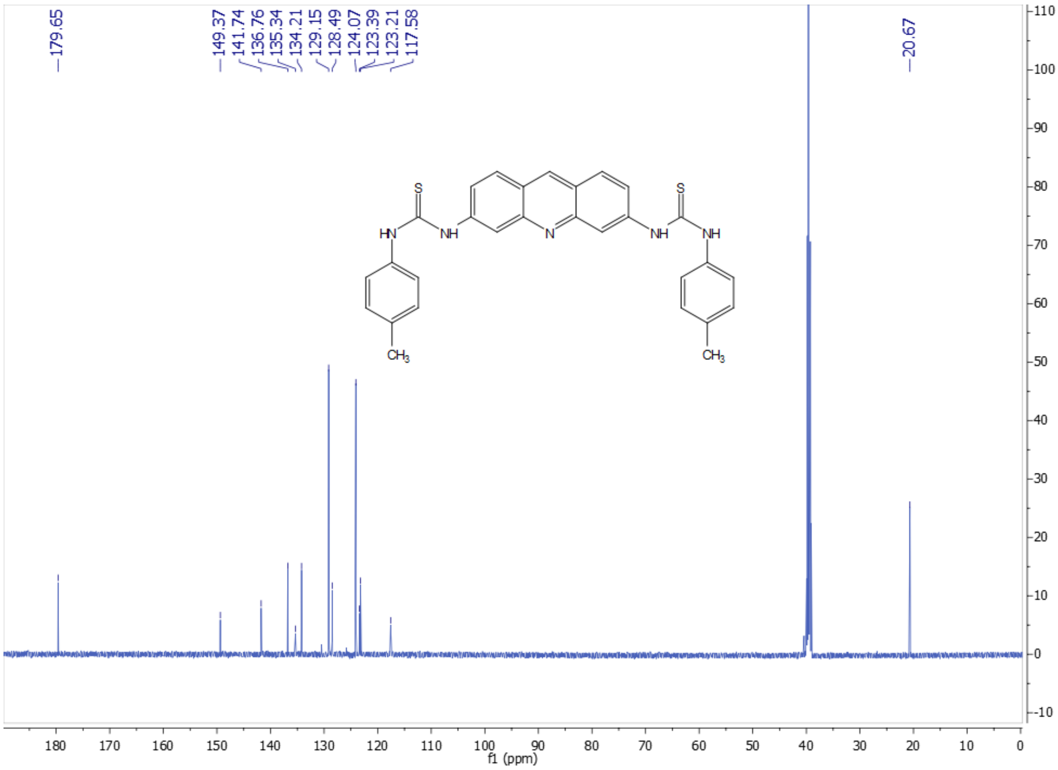

**Figure S12**. $^{13}C$ NMR spectra (DMSO-$d_6$, 150 MHz) of the acridine tiourea **10e**.

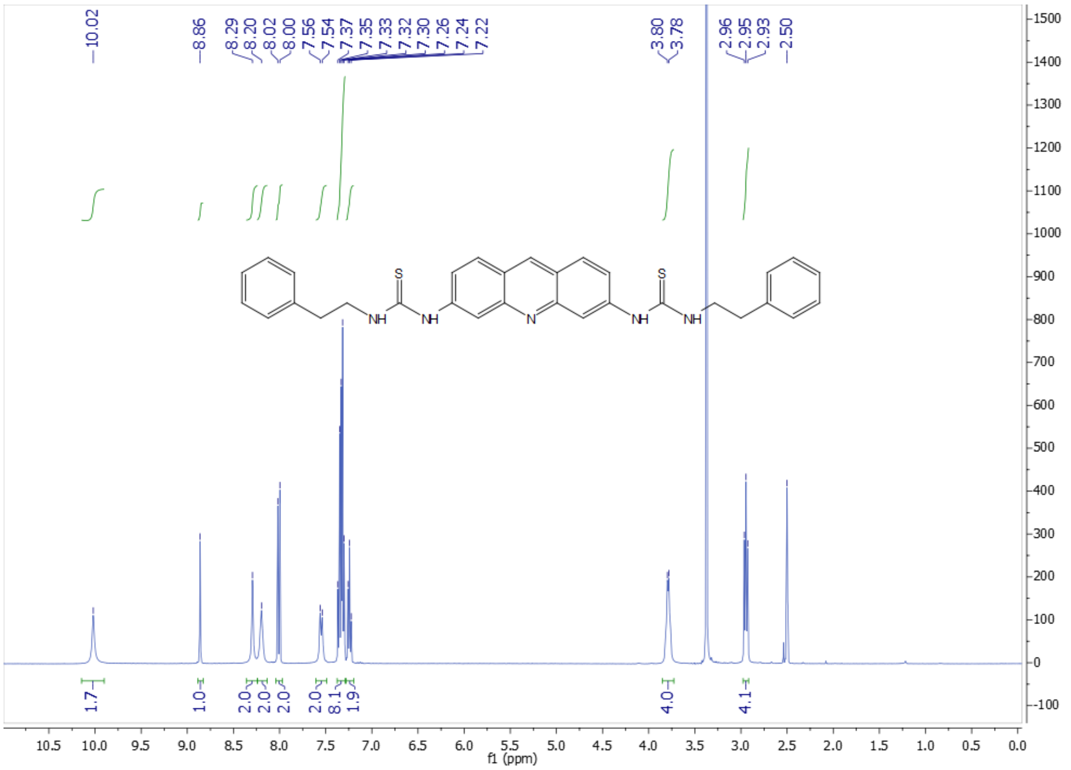

**Figure S13**. $^{1}$H NMR spectra (DMSO-$d_6$, 400 MHz) of the acridine tiourea **10f**.

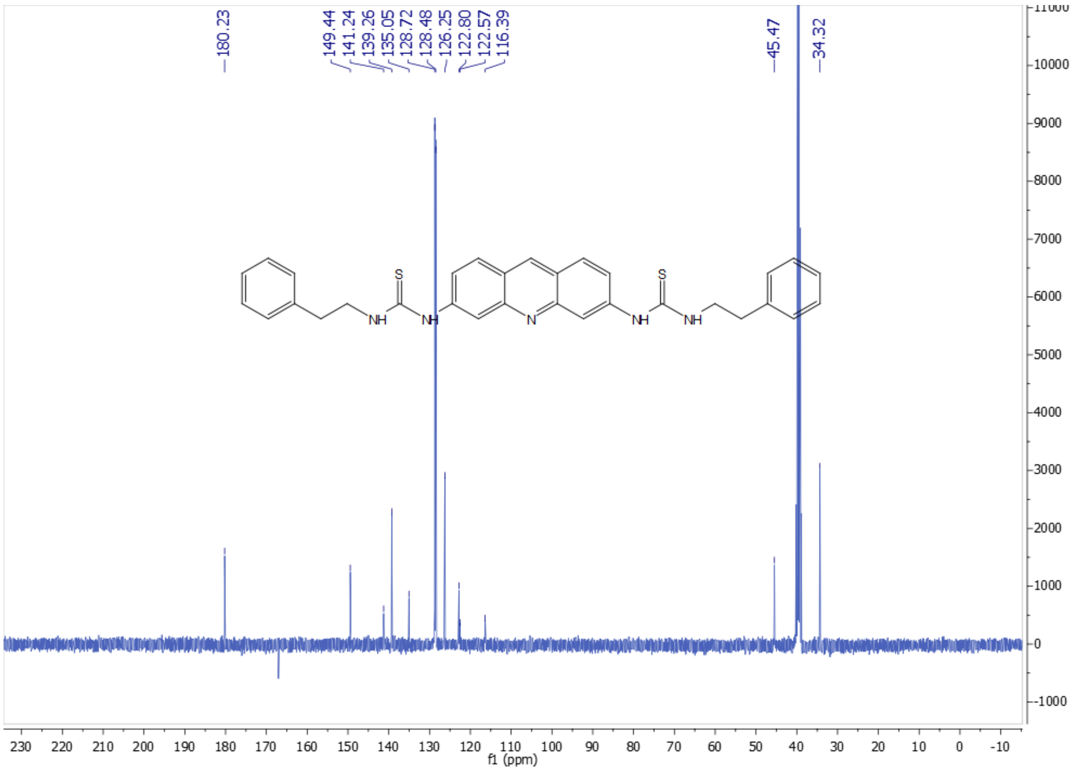

**Figure S14**. $^{13}$C NMR spectra (DMSO-$d_6$, 100 MHz) of the acridine tiourea **10f**.

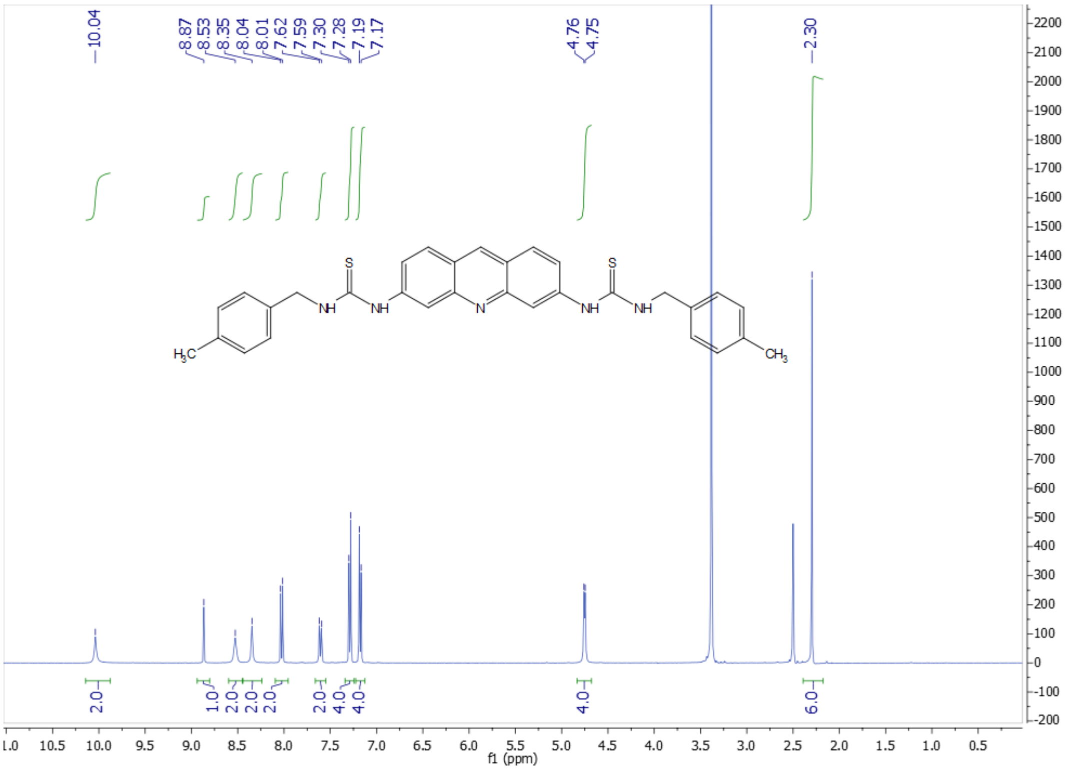


**Figure S15**. $^{1}$H NMR spectra (DMSO-$d_6$, 400 MHz) of the acridine tiourea **10g**.

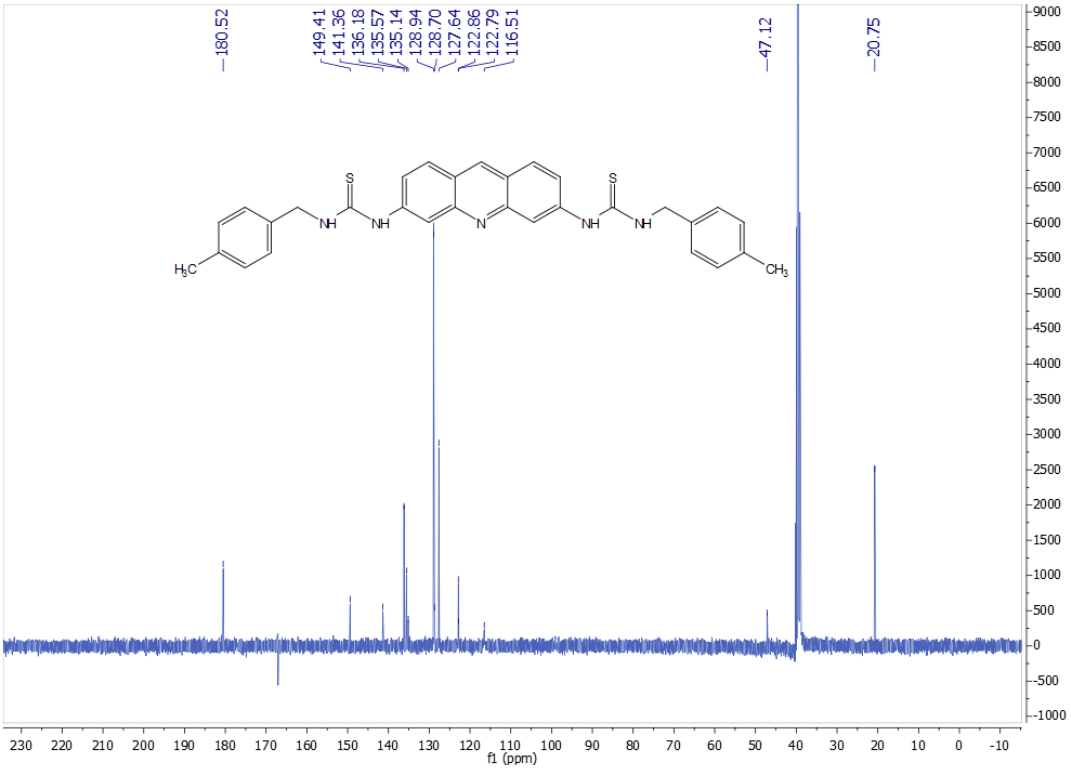


**Figure S16**. [13C] NMR spectra (DMSO-$d_6$, 100 MHz) of the acridine tiourea **10g**.

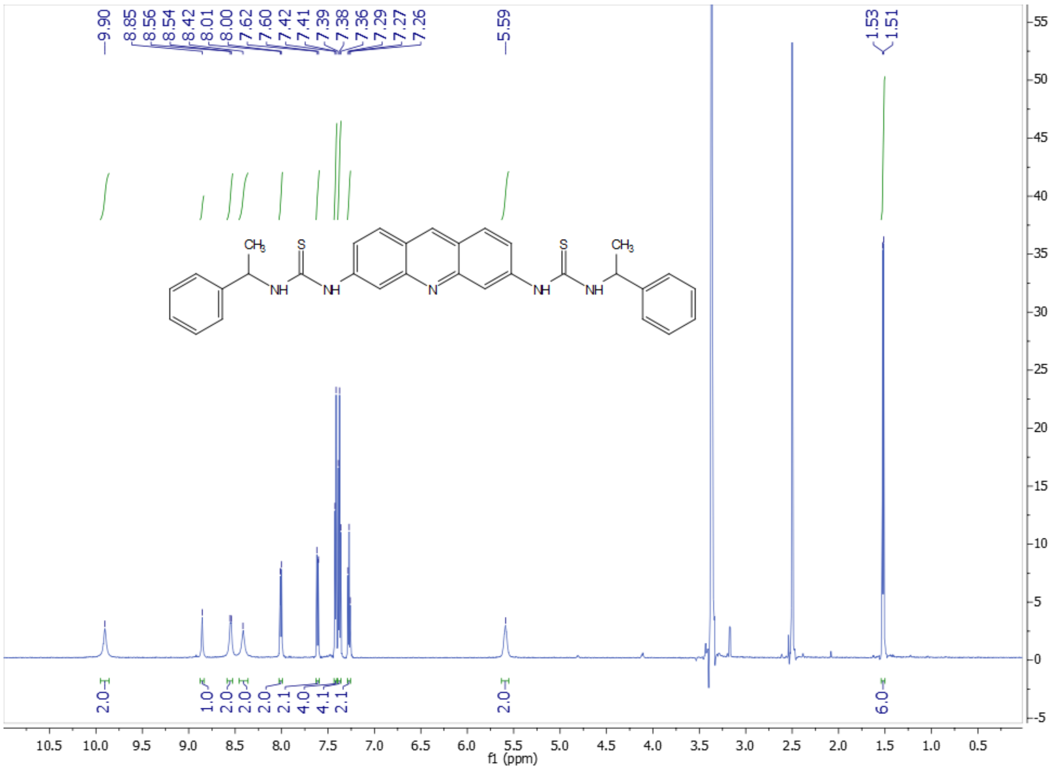


**Figure S17**. $^{1}$H NMR spectra (DMSO-$d_6$, 600 MHz) of the acridine tioureas **10h**,**10i**.

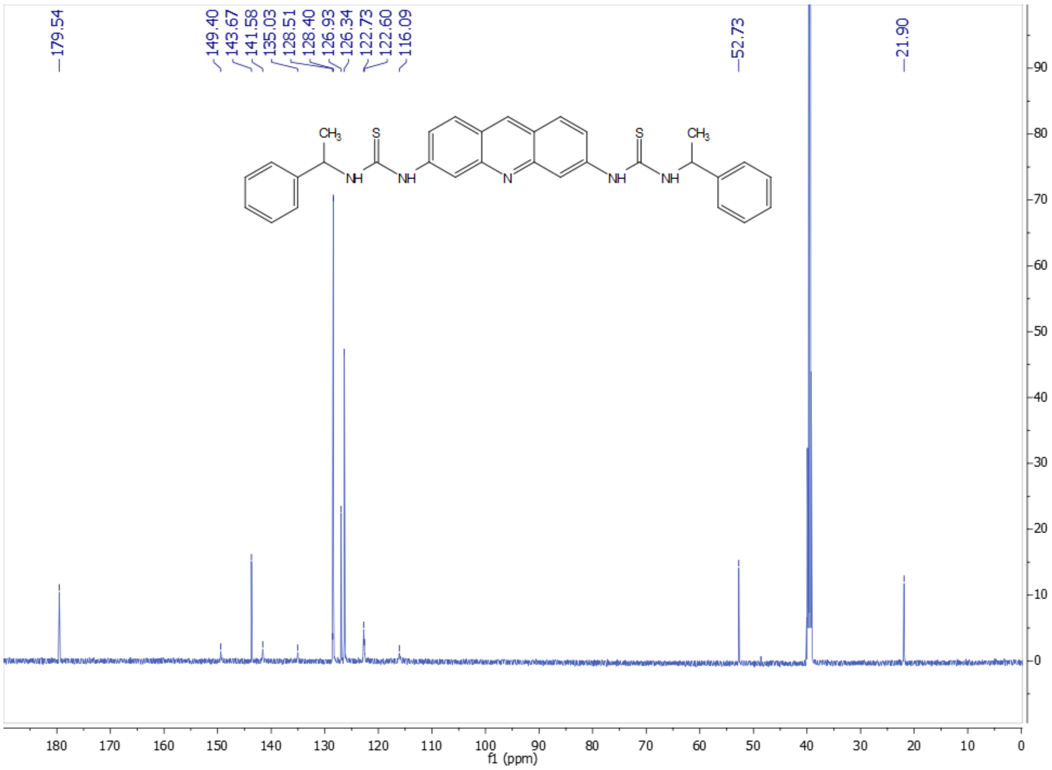


**Figure S18**. $^{13}$C NMR spectra (DMSO-$d_6$, 150 MHz) of the acridine tioureas **10h**,**10i**.

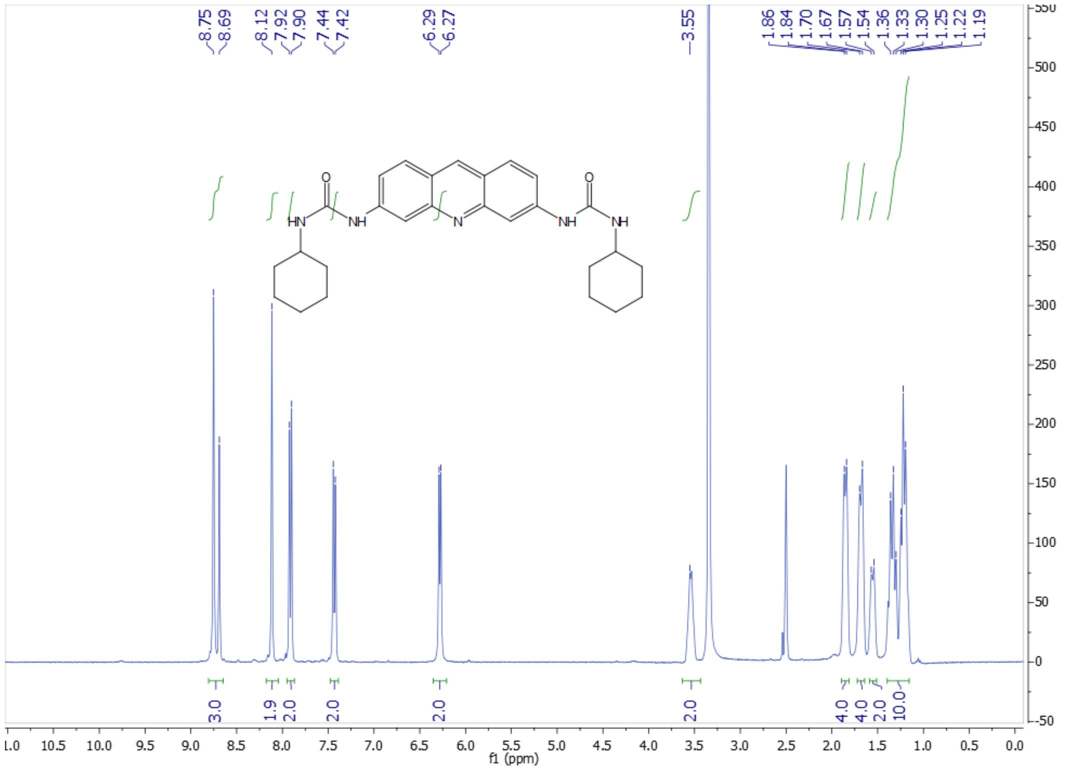

**Figure S19**. $^{1}$H NMR spectra (DMSO-$d_6$, 400 MHz) of the acridine urea **11a**.

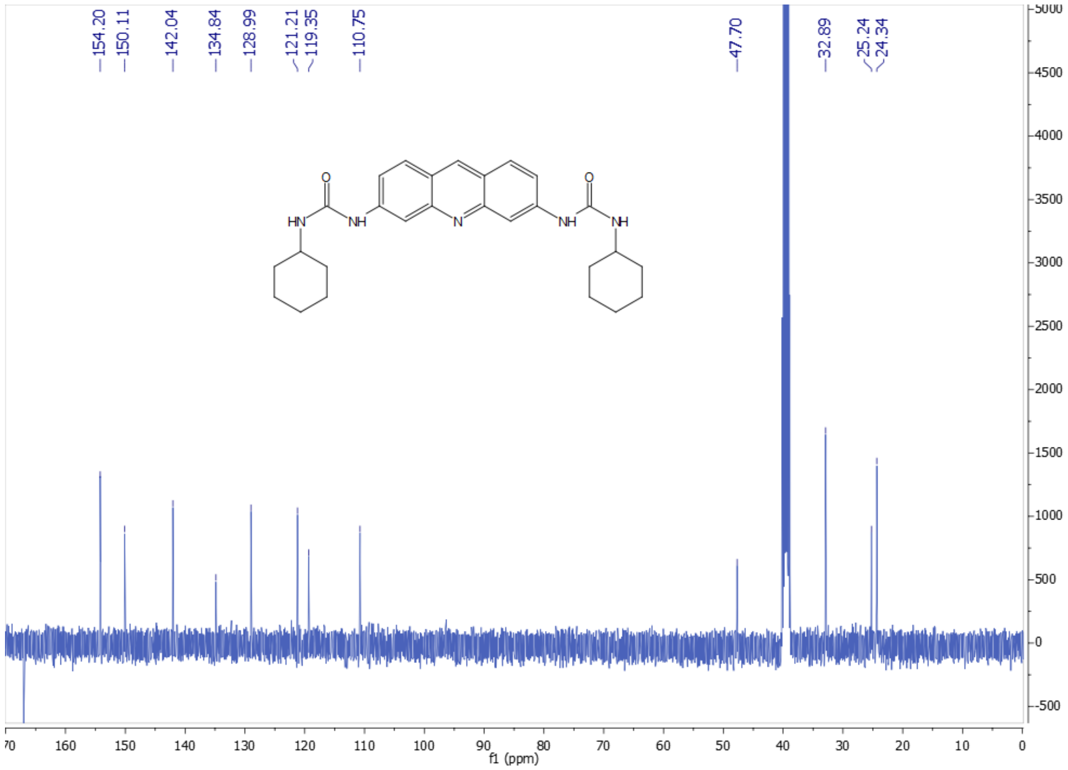

**Figure S20**. $^{13}$C NMR spectra (DMSO-$d_6$, 100 MHz) of the acridine urea **11a**.

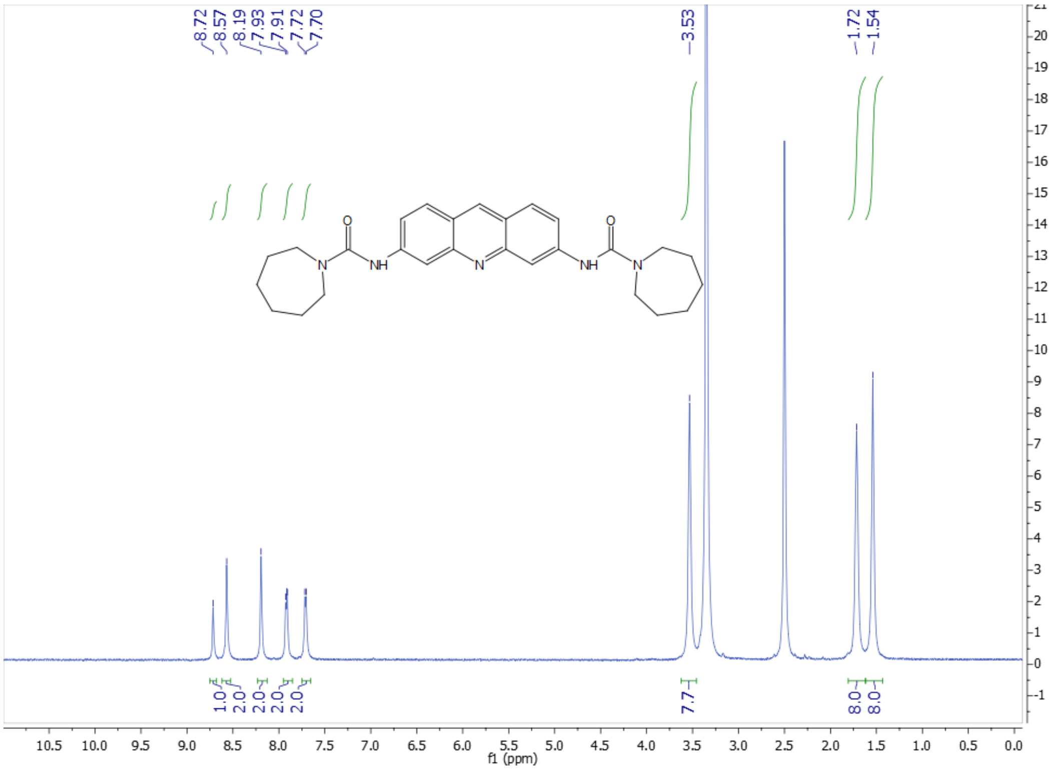

**Figure S21**. $^{1}$H NMR spectra (DMSO-$d_6$, 600 MHz) of the acridine urea **11b**.

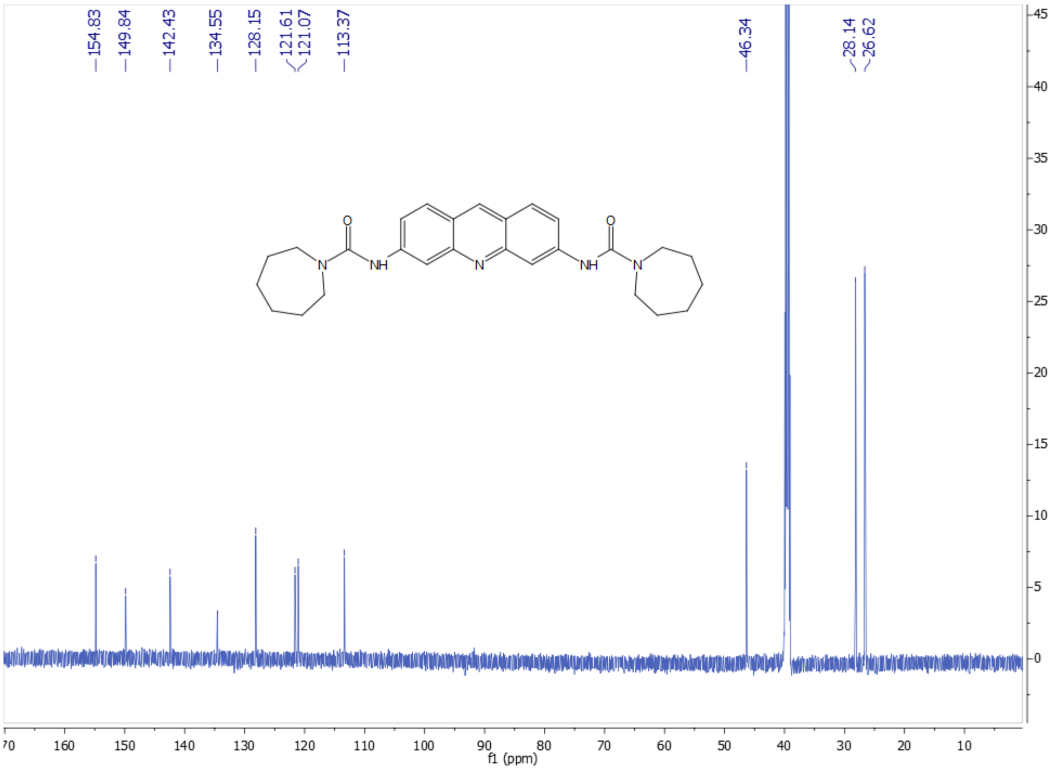

**Figure S22**. $^{13}$C NMR spectra (DMSO-$d_6$, 150 MHz) of the acridine urea **11b**.

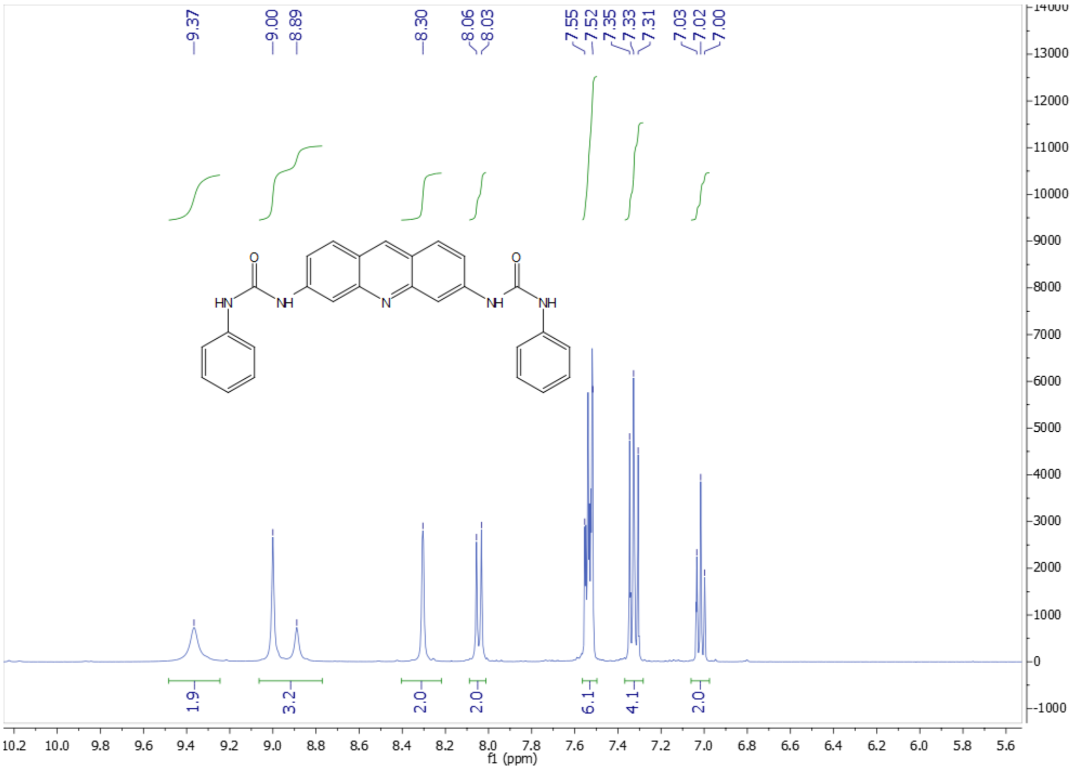


**Figure S23**. $^{1}$H NMR spectra (DMSO-$d_6$, 400 MHz) of the acridine urea **11c**.

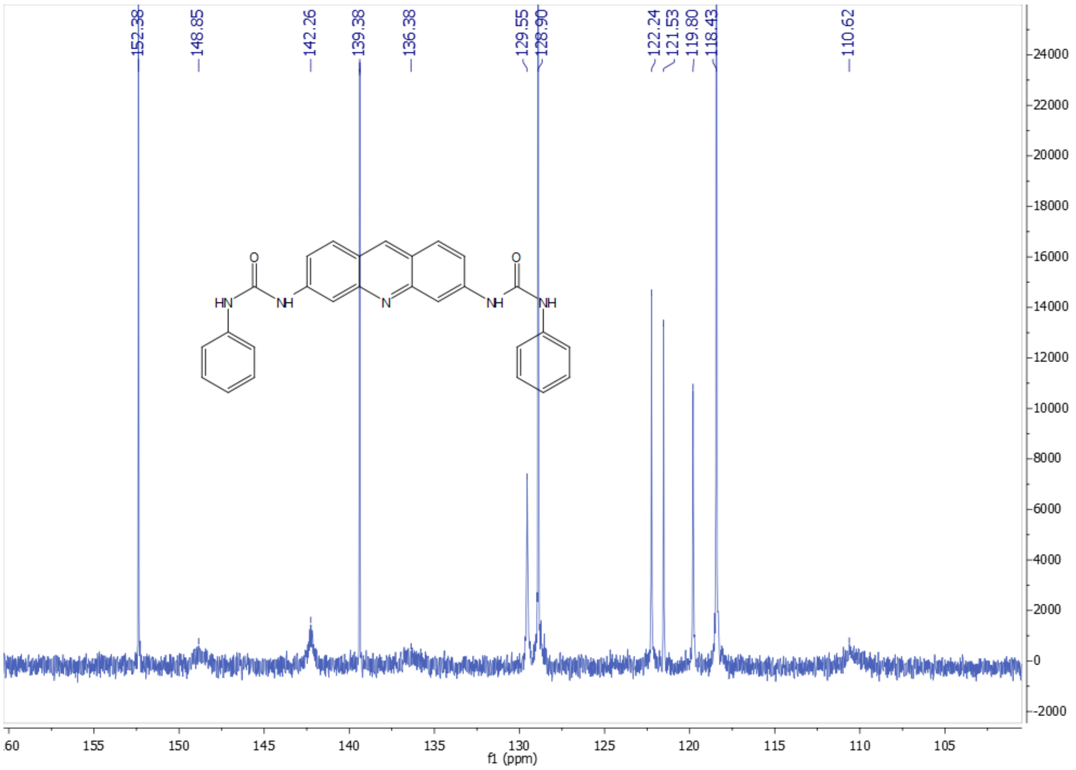


**Figure S24**. $^{13}$C NMR spectra (DMSO-$d_6$, 100 MHz) of the acridine urea **11c**.

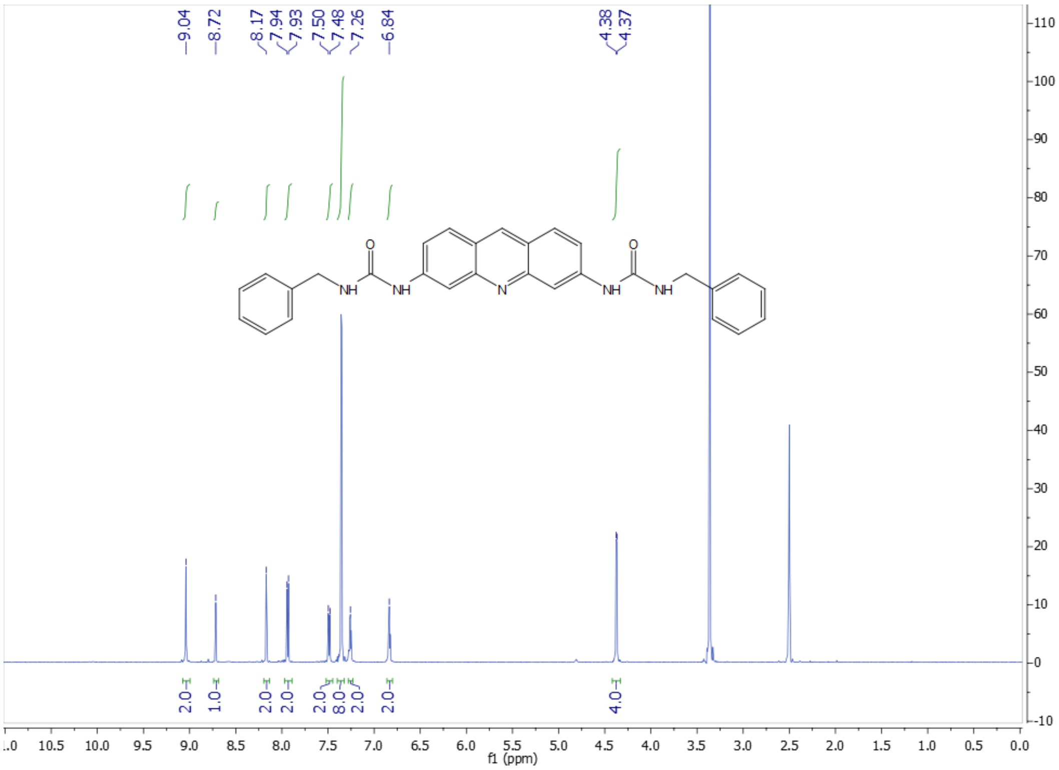


**Figure S25**. $^{1}$H NMR spectra (DMSO-$d_6$, 600 MHz) of the acridine urea **11d**.

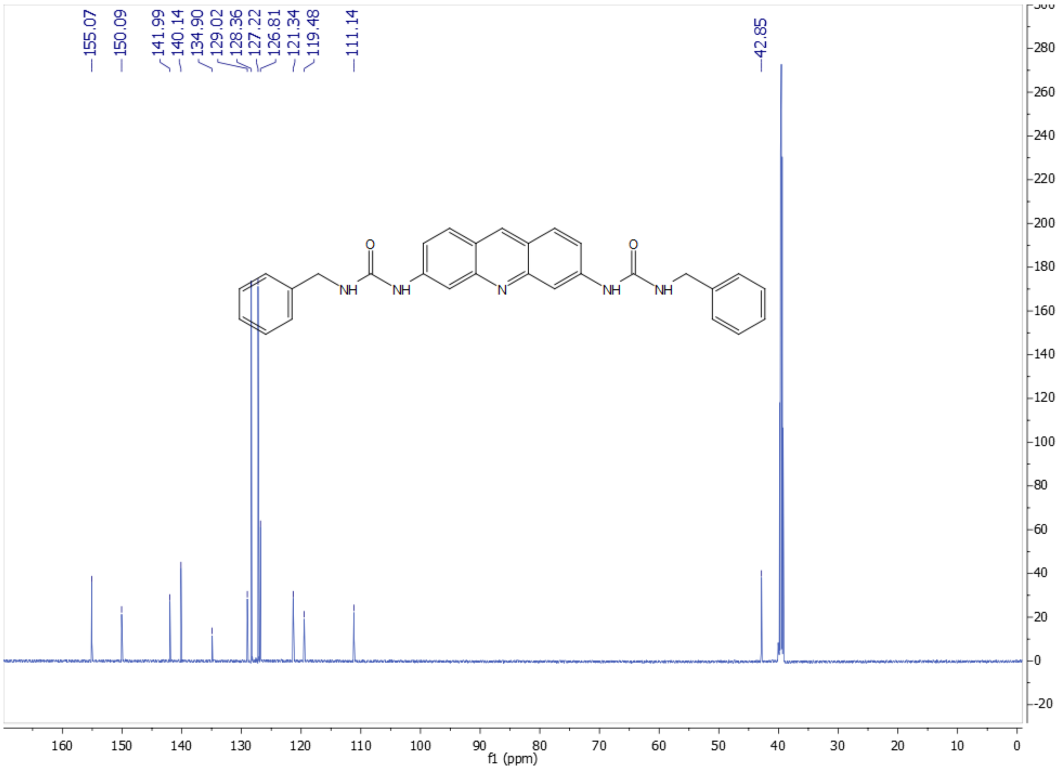


**Figure S26**. $^{13}$C NMR spectra (DMSO-$d_6$, 150 MHz) of the acridine urea **11d**.

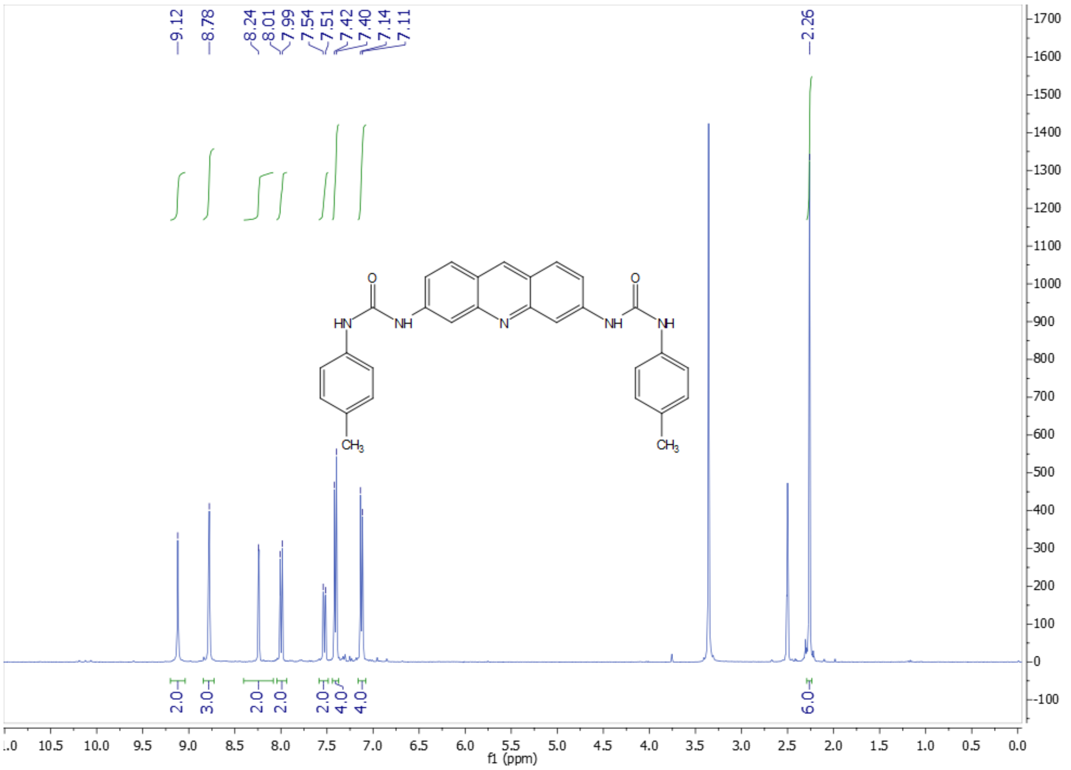

**Figure S27**. $^{1}$H NMR spectra (DMSO-$d_6$, 400 MHz) of the acridine urea **11e**.

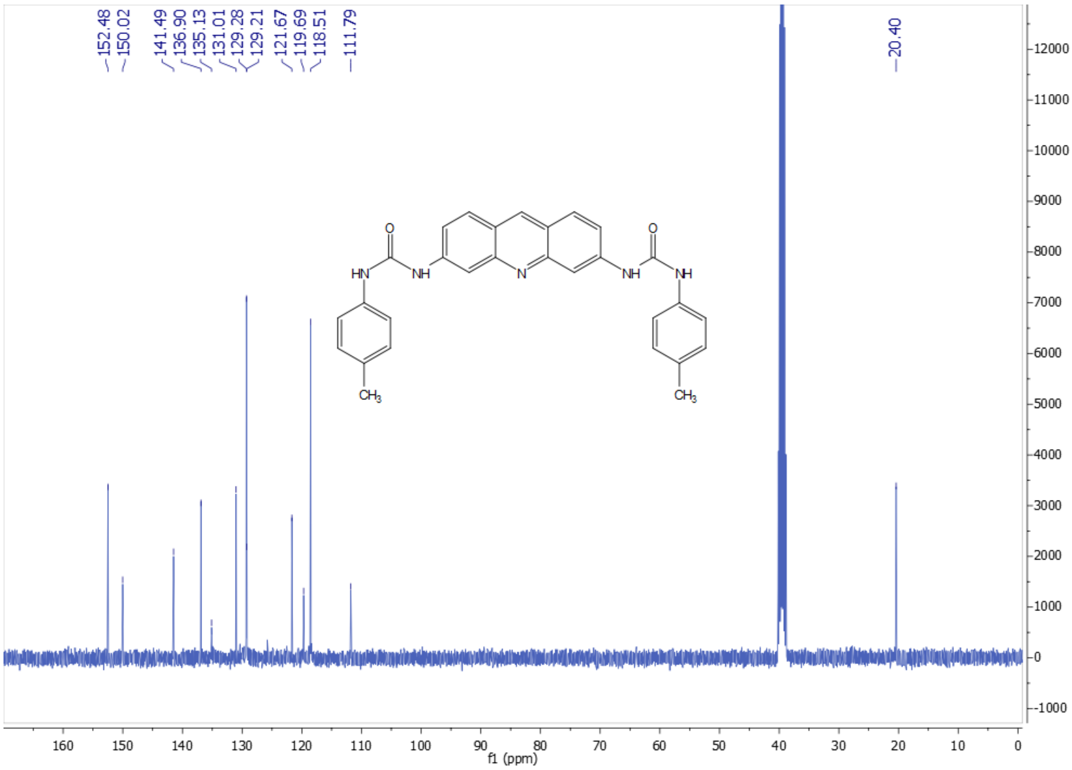

**Figure S28**. $^{13}$C NMR spectra (DMSO-$d_6$, 100 MHz) of the acridine urea **11e**.

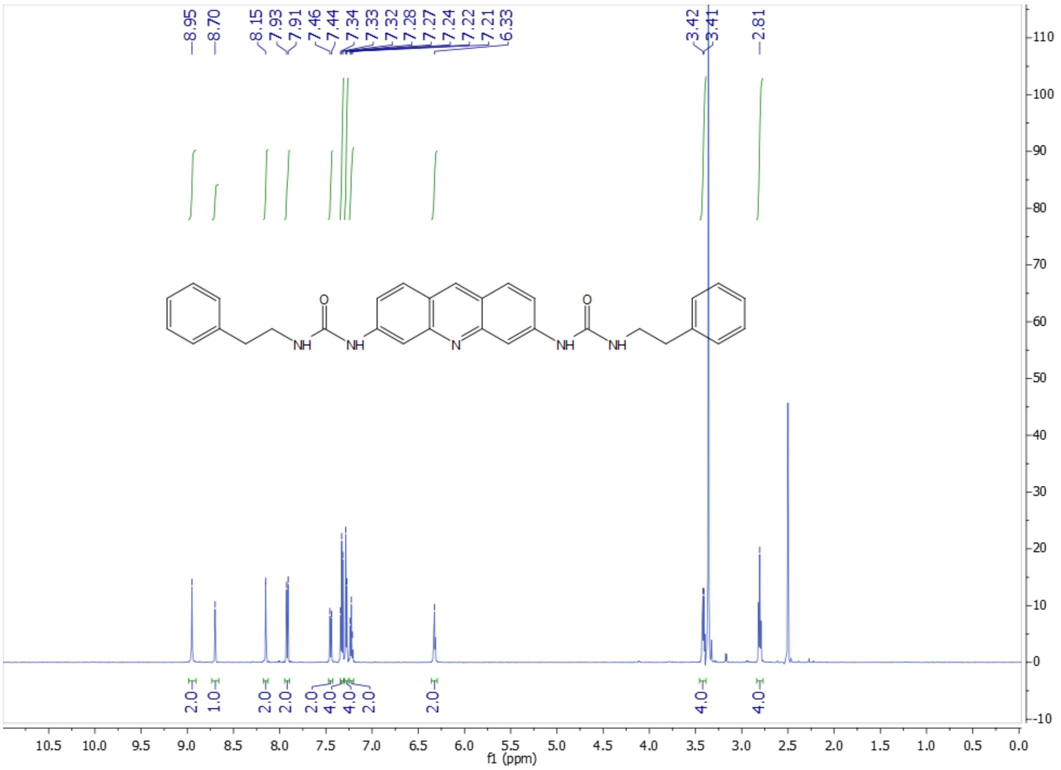


**Figure S29**. $^{1}$H NMR spectra (DMSO-$d_6$, 600 MHz) of the acridine urea **11f**.

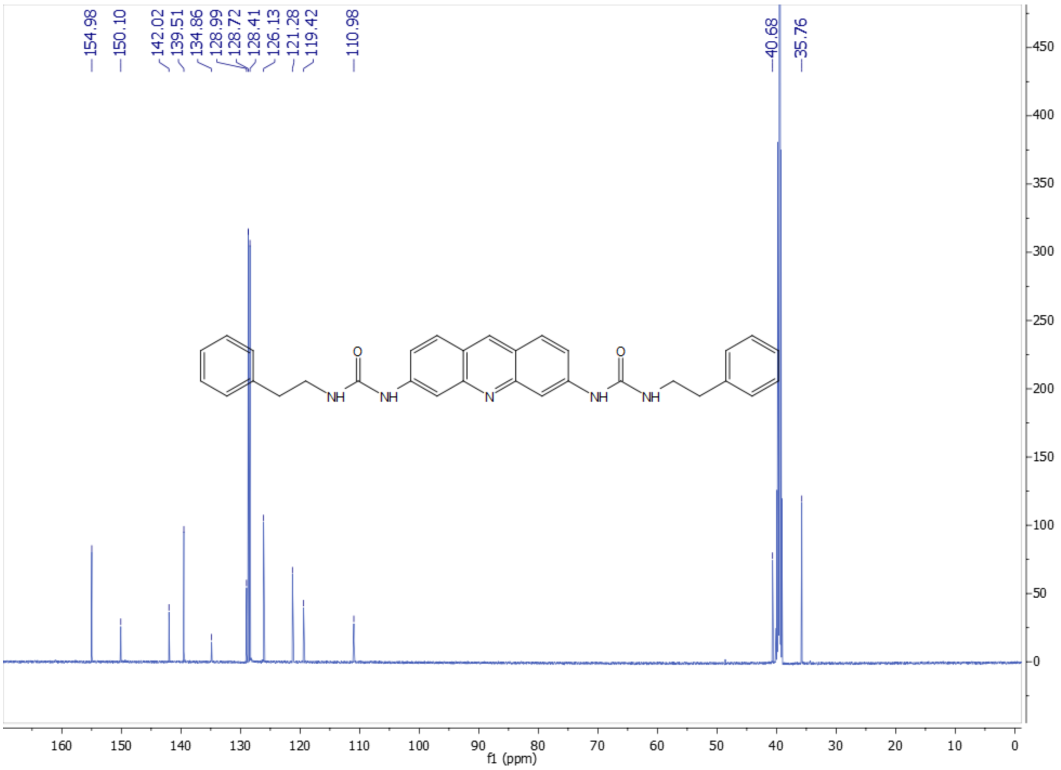


**Figure S30**. $^{13}$C NMR spectra (DMSO-$d_6$, 150 MHz) of the acridine urea **11f**.

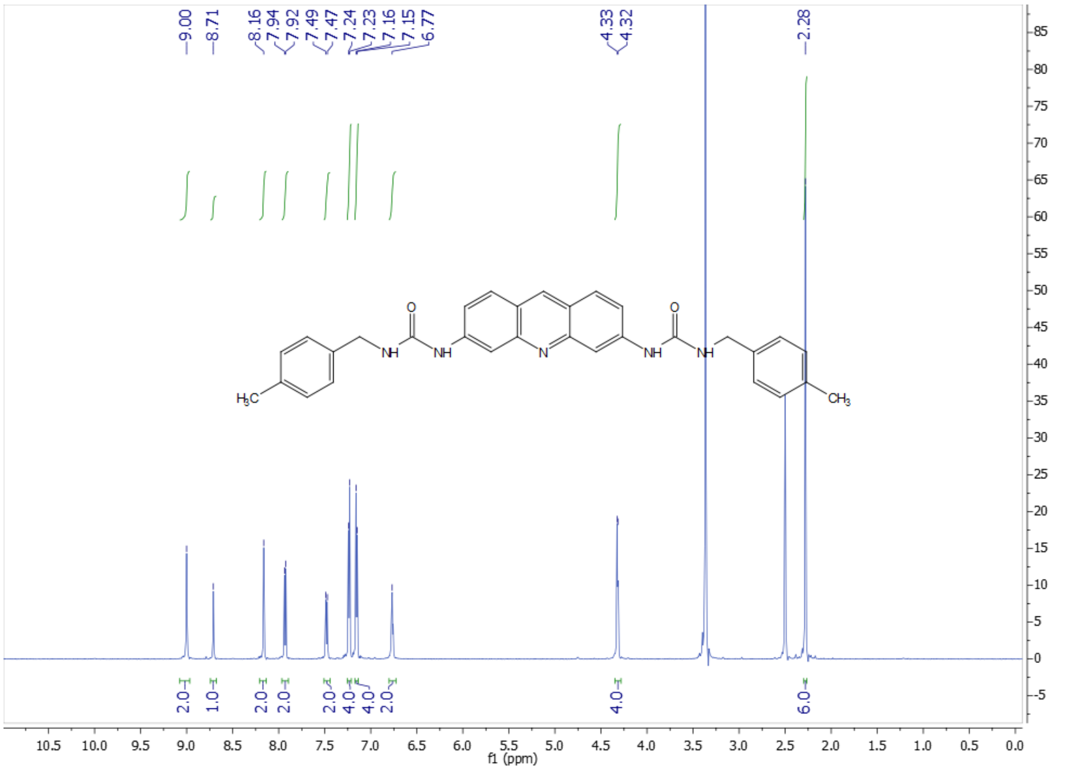

**Figure S31**. $^{1}$H NMR spectra (DMSO-$d_6$, 600 MHz) of the acridine urea **11g**.

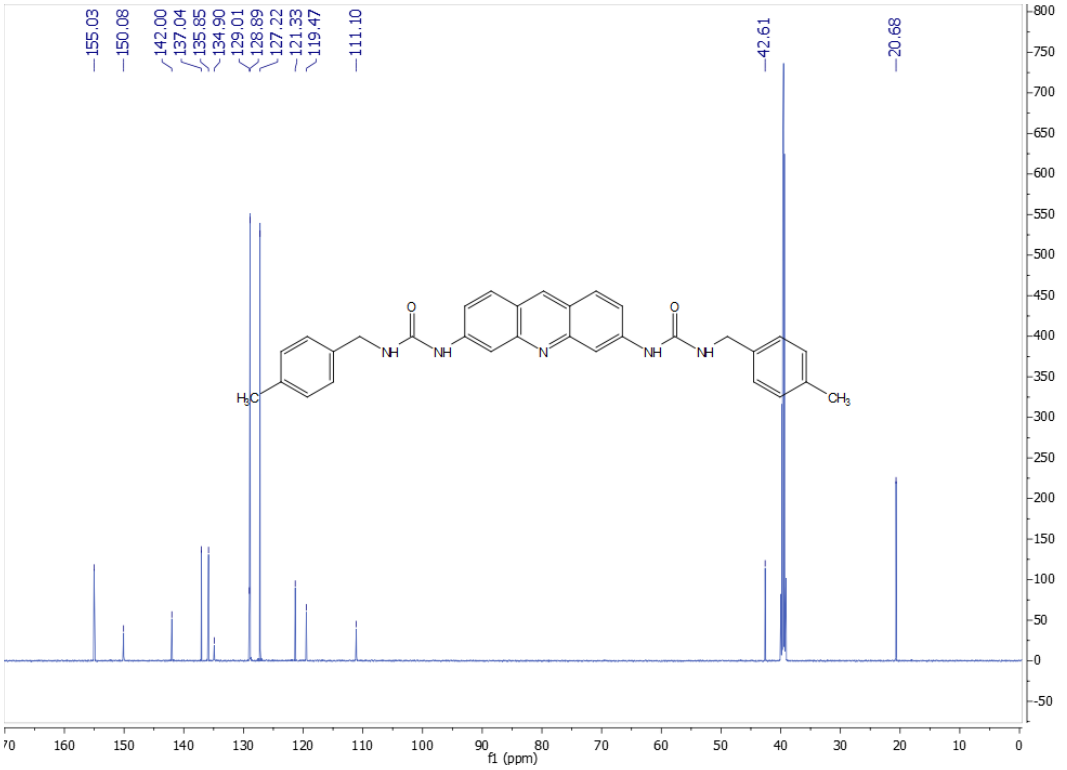

**Figure S32**. $^{13}$C NMR spectra (DMSO-$d_6$, 150 MHz) of the acridine urea **11g**.

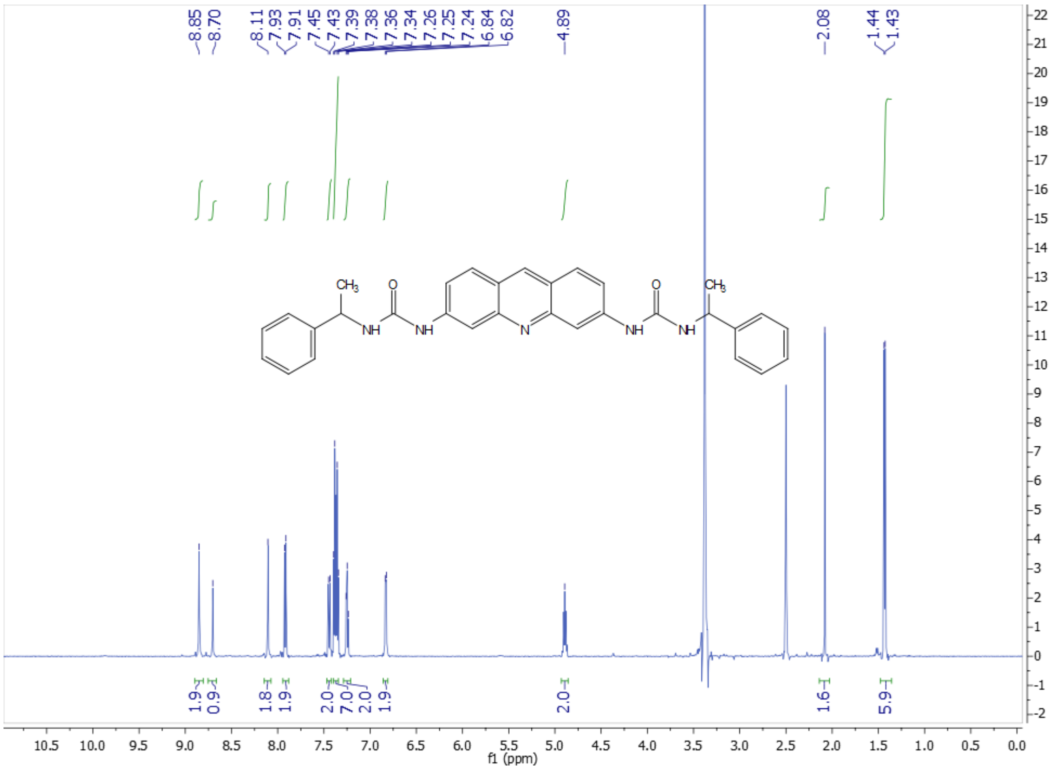

**Figure S33**. $^{1}$H NMR spectra (DMSO-$d_6$, 600 MHz) of the acridine urea **11h**, **11i**.

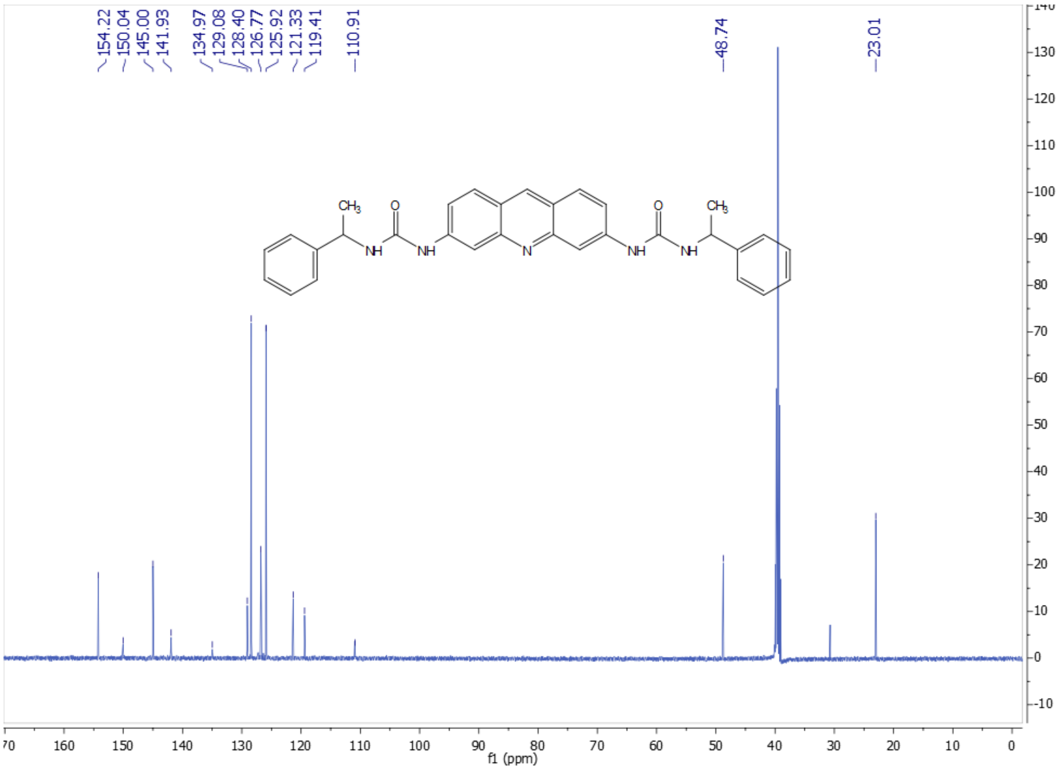

**Figure S34**. $^{13}$C NMR spectra (DMSO-$d_6$, 150 MHz) of the acridine urea **11h**, **11i**.
